# Direct Evidence of Microbial Sunscreen Production by Scum‐Forming Cyanobacteria in the Baltic Sea

**DOI:** 10.1111/1758-2229.70056

**Published:** 2025-01-16

**Authors:** Inkeri Vuori, Greta Gaiani, Sıla Arsın, Endrews Delbaje, Julia Järn, Robert Snårbacka, Annaël Couëdelo, Gayathri Murukesan, Matti Wahlsten, Jouni Jokela, Tânia Keiko Shishido, David P. Fewer

**Affiliations:** ^1^ Department of Microbiology University of Helsinki Helsinki Finland; ^2^ Universidade de São Paulo Center for Nuclear Energy in Agriculture Piracicaba Brazil

**Keywords:** Baltic Sea, bloom, cyanobacteria, mycosporine‐like amino acids, phylogenomic analysis, porphyra‐334, shinorine

## Abstract

Mycosporine‐like amino acids are water‐soluble secondary metabolites that protect photosynthetic microorganisms from ultraviolet radiation. Here, we present direct evidence for the production of these compounds in surface scums of cyanobacteria along the Baltic Sea coast. We collected 59 environmental samples from the southern coast of Finland during the summers of 2021 and 2022 and analysed them using high‐resolution liquid chromatography‐mass spectrometry. Our results revealed the presence of microbial sunscreens in nearly all surface scum samples. Mycosporine‐like amino acids are synthesised through the coordinated action of four biosynthetic enzymes encoded in a compact biosynthetic pathway. Bioinformatics analysis of the mysB biosynthetic gene from a surface scum indicated that the cyanobacteria responsible for production belonged to the *Anabaena*/*Dolichospermum*/*Aphanizomenon* species complex. We mapped the distribution of biosynthetic enzymes onto a phylogenomic tree, utilising 120 bacterial single‐copy conserved genes from 101 draft or complete genomes within the species complex. This analysis showed that 48% of identified species possess the ability to produce these compounds, with biosynthetic pathways being most common in *Dolichospermum* and *Aphanizomenon* strains. We detected the production of porphyra‐334 and shinorine, two widely reported family members, in *Dolichospermum* strains isolated from the Gulf of Finland's surface layer. The estimated content of porphyra‐334 in *Dolichospermum* sp. UHCC 0684 was 7.4 mg per gram dry weight. Our results suggest that bloom‐forming cyanobacteria could be a potential source of these compounds for cosmetic and biotechnological applications and may play a significant role in cyanobacterial bloom formation.

## Graphical abstract

We found both culture‐independent and culture‐dependent evidence for the widespread production of mycosporine‐like amino acids (MAAs) in blooms of cyanobacteria from the Baltic Sea. Using a combination of bioinformatics, microbial genetics, and mass spectrometry, we demonstrated the high‐level production of these microbial sunscreens in members of the *Anabaena*/*Dolichospermum*/*Aphanizomenon* (ADA) species complex. Our results provide new insights into the ecological role of MAAs in the formation of surface blooms of cyanobacteria.

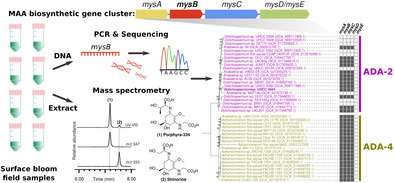

## 1 Introduction

Photosynthetic organisms depend on sunlight and are exposed to harmful ultraviolet radiation (Gao and Garcia‐Pichel 2011a, 2011b). Ultraviolet radiation adversely affects organisms through damaging photochemical reactions and oxidative stress (Cockell and Knowland 1999; Shick and Dunlap 2002). Many photosynthetic organisms produce photoprotective compounds that dissipate the energy of harmful ultraviolet radiation (Llewellyn and Harbour 2003; Gao and Garcia‐Pichel 2011a, 2011b; Geraldes et al. 2020). Mycosporine‐like amino acids (MAAs) are a family of colourless, polar, uncharged, water‐soluble secondary metabolites with a low molecular weight (Gao and Garcia‐Pichel 2011a, 2011b). They contain either a cyclohexenone or a cyclohexenimine chromophore, and a variety of nitrogen containing substituents (Wada, Sakamoto, and Matsugo 2013). MAAs have absorption maxima that fall within the range of harmful UV‐A (315–400 nm) and UV‐B (280–315 nm) and their high molar extinction coefficients enable the efficient absorption of ultraviolet radiation (Gao and Garcia‐Pichel 2011a, 2011b; Geraldes and Pinto 2021). Uniquely, MAAs dissipate this energy as heat without creating harmful photoproducts, such as reactive oxygen species (Gao and Garcia‐Pichel 2011a, 2011b). Some MAA variants have also been shown to function as antioxidants (Wada, Sakamoto, and Matsugo 2015) and are believed to provide protection against oxidative stress (Oren and Gunde‐Cimerman 2007). MAAs are reported from fungi, cyanobacteria, eukaryotic algae, and zooplankton (Sinha, Singh, and Hader 2007). There are 74 chemical variants reported to date from the MAA family, with porphyra‐334 and shinorine being the most frequently reported (Llewellyn and Airs 2010; Jain et al. 2018; Geraldes and Pinto 2021).

MAAs are synthesised through the shikimate pathway or pentose phosphate pathways in cyanobacteria where the biosynthetic enzymes MysA–MysD are encoded in compact biosynthetic gene clusters (Balskus and Walsh 2010; Gao and Garcia‐Pichel 2011a; Dittmann et al. 2015). The biosynthetic steps leading to the formation of shinorine and porphyra‐334 have been elucidated through a series of genetic and biochemical studies (Balskus and Walsh 2010; Gao and Garcia‐Pichel 2011a; Pope et al. 2015; Chen et al. 2021). The MysA and MysB enzymes are responsible for the biosynthesis of the 4‐deoxygadusol chromophore shared by all MAA variants (Balskus and Walsh 2010). The biosynthetic precursors of the MysA enzyme are sedoheptulose‐7‐phosphate from the pentose phosphate pathway or 3‐dehydroquinate from the shikimate pathway (Balskus and Walsh 2010; Pope et al. 2015). The MysC and MysD enzymes are responsible for the attachment of a wide variety of amino acid substituents to the chromophore (Balskus and Walsh 2010; Gao and Garcia‐Pichel 2011a).

Cyanobacterial blooms are a recurring phenomenon in the Baltic Sea where they can form surface scums due to factors that include low turbulence levels, high water temperatures, gas bubbles, and the buoyant capacity of cyanobacterial cells (Medrano et al. 2016). Eutrophication combined with warm temperatures and long period of sunlight promotes the development of cyanobacterial blooms (Finni et al. 2001). The Baltic Sea is a brackish water body, with low salinity and limited water exchange, that represents the perfect conditions to thrive for bloom‐forming cyanobacteria as *Nodularia spumigena* and *Aphanizomenon flos‐aquae* (Beltran‐Perez and Waniek 2021). Even though scum formation is a poorly understood process, scums of cyanobacteria are exposed to harmful UV radiation (Medrano et al. 2016) and the MAA biosynthetic pathway is reported from many bloom‐forming cyanobacteria (Subramaniam et al. 1999; Liu, Häder, and Sommaruga 2004; Zhang et al. 2022). Furthermore, previous studies demonstrate that bloom‐forming cyanobacteria produce MAAs in culture and in lakes (Geraldes et al. 2020; Zhang et al. 2022; Sommaruga, Chen, and Liu 2009; Hu et al. 2018; Tartarotti and Sommaruga 2006). Therefore, cyanobacteria forming blooms and surface scums during summer, exposed to intense UV radiation, could be a natural reservoir for MAAs.

In this study, we undertook an extensive two‐year sampling effort along the southern coast of Finland to explore cyanobacterial blooms as potential natural reservoirs of MAAs. High‐resolution liquid chromatography mass spectrometry (LC–MS) analysis confirmed the presence of MAAs, including porphyra‐334 and shinorine, in 59 environmental samples collected in 2021 and 2022. We also detected the *mysB* gene, a key gene in the MAA biosynthetic pathway, in all samples, providing further evidence of MAA production potential. Sequencing data from one sample revealed that the dominant cyanobacteria belonged to the ADA species complex. Extended bioinformatic analyses also revealed prevalence of MAA biosynthetic gene clusters in ADA species complex, specifically in Clades 2 and 4. In addition, previous Baltic Sea bloom isolates that belong to ADA clades which had been maintained in the laboratory culture collection were also screened for MAA production and revealed that *Dolichospermum* sp. UHCC 0684 and UHCC 0260 were high porphyra‐334 producers.

## 2 Materials and Methods

### 2.1 Field Sampling of Naturally Occurring Blooms

The transient nature of cyanobacterial blooms makes it difficult to collect samples from surface scums (Finni et al. 2001) because predicting when and where an event will occur is a complicate task. For this reason, sampling sites were identified using reports using the Finnish public up‐to‐date water information database on https://www.vesi.fi/. The website was consulted daily for the whole duration of the sampling campaign (June–September 2021 and 2022). We collected 59 samples of surface blooms from the water bodies in southern Finland (Table S1). The samples were transported to the laboratory and transferred to a 1 or 2 L graduated cylinder according to sample volume and let to settle overnight at 4°C. A sample of the cyanobacteria accumulating on the surface was collected for microscopic analysis using a Zeiss Axioskop 2 plus Light Microscope (Jena, Germany). Light photomicrographs were obtained using a Zeiss Axiocam 305 colour camera and further processed using Zeiss ZEN Blue 3.1. One mL of the collected biomass was transferred to a 1.5 mL Eppendorf tube and stored at −20°C prior to DNA analysis. The remaining biomass was transferred to 0.5 L plastic boxes (Orthex Oy Ab, Lohja) and stored at −20°C prior to MC analysis.

### 2.2 DNA Extraction and PCR Amplification

40 mg from the sample stored for DNA extraction at −20°C were homogenised at room temperature using a FastPrep‐24 cell disrupter (MP Biomedicals, Santa Ana, CA, USA) two times for 20 s at a speed of 6.5 m s^−1^. DNA extraction was performed starting from 200 μL of each bloom sample and using DNeasy PowerSoil Pro kit (Qiagen, GmbH, Hilden, Germany) following manufacturer's instruction. The quantity and quality of the extracted DNA were estimated using a NanoDrop One/OneC Microvolume UV–Vis Spectrophotometer (Thermo Scientific). Partial 16S rRNA gene sequences were amplified by PCR using the cyanobacteria‐specific primers Cya359F 5′‐GGGGAATYTTCCGCAATGGG‐3′ and Cya781R 5′‐GACTACWGGGGTATCTAATCCCWTT‐3′ (Nübel, Garcia‐Pichel, and Muyzer 1997) to check for the presence of PCR inhibitors in the extracted environmental DNA and to verify the presence of cyanobacteria. The PCR reactions were prepared in 25 μL aliquots containing 2.5 μL of 10 × DreamTaq Green buffer, 0.2 mM of each deoxynucleotide triphosphates (dNTPs; Thermo Scientific, Waltham, MA, USA), 0.5 μΜ of each of the oligonucleotide primers (Sigma‐Aldrich, St. Louis, MI, USA), 70–80 ng of environmental DNA and 1.25 U of DreamTaq DNA Polymerase (5 U/μL). The PCR reactions were run on a SimpliAmp Thermal Cycler (ThermoFisher Scientific) with an initial denaturation step at 94°C for 4 min followed by 30 cycles consisting of 94°C for 1 min, 55°C for 1 min, and 72°C for 1 min followed by a 7 min extension step at 72°C as previously described (Christodoulou, Meletiou‐Christou, and Parmakelis 2015). The resulting PCR products were separated on a 0.8% (w/v) agarose gel containing 0.5 × TAE stained with a 0.625 mg mL^−1^ ethidium bromide (Thermo Scientific, Waltham, MA, USA) at 120 V for 20–25 min and visualised for the presence or absence of PCR products of the expected length using a UV transilluminator.

### 2.3 Analysis of the Cyanobacterial Cultures From University of Helsinki Culture Collection

Thirty‐six strains of *Dolichospermum* that were previously isolated from the surface layer of the Gulf of Finland (Halinen et al. 2007, 2008) were selected to evaluate the production of MAAs (Table S4). The isolated cultures are available from the University of Helsinki Culture Collection (UHCC) and are maintained in 40 mL of sterile Z8 medium (Kotai 1972) lacking a nitrogen source, and alternatively, supplemented with NaCl and MgSO₄ (Table S4). They were cultivated under 5 μmol of constant light intensity at 17°C for 4 weeks. Biomass from 40 mL of the isolates was harvested by centrifugation of 7000×g for 7 min at 20°C and pelleted cells were freeze‐dried as described above. Then, for MAAs extraction, 10 mg of freeze‐dried biomass from each cyanobacterial culture were used and processed in the same way as previously described.

### 2.4 DNA Extraction and Genome Sequencing

Genomic DNA from *Dolichospermum* spp. UHCC 0260 and UHCC 0684 were extracted using DNeasy PowerSoil kit (QIAGEN, Netherlands) according to the manufacturer's instructions as previously described (Österholm et al. 2020). Whole genome sequencing was performed using Illumina MiSeq and the quality of the Illumina reads was checked by running FastQC v0.10.1. Adapter contaminations were removed using Cutadapt 1.18 (Martin 2011) and further trimming was performed with Prinseq v0.20.4 (Schmieder and Edwards 2011). Genome assembly was performed using Spades v3.15.4 using default parameters (Prjibelski et al. 2020). Kaiju 1.8.2 was used to improve the quality of the assembly which was then evaluated with Quast v5.0.2 (Gurevich et al. 2013). We used CheckM 1.1.3 to calculate genome completeness and contamination (Parks et al. 2015). The coverage of the assembled genome was calculated by mapping the reads with Bowtie2 v2.4.5. We used Bedtools v2.27.1 to calculate the sequence coverage. Draft genome sequences were deposited in NCBI.

### 2.5 Screening of Bloom Samples for MAA Biosynthetic Gene Cluster Using the *mysB* Gene

We designed the oligonucleotide primer pair MysBF (5′‐GCTTTCTGGACATATKGAAGG‐3′) and MysBR (5′‐GATGATGGTTAAACCRTCTCG‐3′) (Eurofins Genomics) to detect the presence of *mysB* gene in the scum samples. This primer pair was used to amplify the *mysB* gene from environmental DNA extracted from sample no. 4 from 2021 (Table S1). To minimise the risk of amplifying non‐*mysB* methyltransferase and the occurrence of false positives, in silico analyses were performed to verify the specificity of the primers for the target region. The PCR reaction was performed according to the manufacturer's instructions and contained, 2.5 μL of 10× DreamTaq TM buffer (ThermoFisher Scientific), 2 μL of 0.2 mM of each dNTPs (Thermo Scientific, Waltham, MA, USA), and 0.5 μΜ of each of the oligonucleotide primers MysBF/MysBR, 30–70 ng environmental DNA and 1.25 U of DreamTaq DNA Polymerase (5 U/μL, ThermoFisher Scientific). Thermocycling conditions were optimised, and the following were selected to achieve amplification: initial denaturation of 95°C for 1 min, 30 cycles consisting of denaturation at 95°C for 30 s, annealing at 55°C for 30 s and extension at 72°C for 1 min, with a final extension at 72°C for 10 min. A PCR product of the expected length was observed in an 0.8% agarose gel separated by gel electrophoresis at 120 V for 20–25 min. The band was excised using a sterile scalpel under UV irradiation and the DNA was purified using the NucleoSpin Gel and PCR Clean‐up (Macherey‐Nagel). The purified PCR product was cloned using the pCR 2.1 vector TA cloning kit (ThermoFisher Scientific) and transformed to OneShot INVαF' chemically competent cells (ThermoFisher Scientific) according to the manufacturer's instructions. The plasmid containing the *mysB* insert was purified using PureLink Quick Plasmid Miniprep kit (ThermoFisher Scientific). The plasmid was Sanger sequenced using the following protocol: the DNA was purified using MultiScreen PCR 96 (Cat No. LSKMPCR50, Merck Millipore) and the sequencing reaction was performed following the manufacturer's instructions using the BigDye Terminator v3.1 Cycle Sequencing Kit (Part No. 4336921, Applied Biosystems). The sequencing reactions were analysed using an ABI3130XL Genetic Analyser (Applied Biosystem) and sequenced using an ABI Prism 3500 (Applied Biosystems). The assembled sequence was obtained using Phred‐Phrap/Consed analyses tools (Machado et al. 2011). The amino acid sequence was obtained using Expasy Translate and the sequence was used to construct a MysB phylogenetic tree. The genomes were annotated with Prokka v1.14.6 (Seemann 2014) and the MAA cluster proteins were identified with BLASTp 2.8.1+ (Camacho et al. 2009) using the amino acid sequences from *Dolichospermum* spp. UHCC 0260 and UHCC 0684 as references. The maximum‐likelihood phylogenetic tree based on the identified MAA proteins were done by aligning the amino acid sequences with MUSCLE (Edgar 2004) and inferring the trees with FastTree 2.1.11 (Price et al. 2010) using the WAG+GAMMA model. The tree was visualised and edited with iTOL (Letunic and Bork 2019).

### 2.6 Phylogenomic Analysis

A maximum‐likelihood phylogenomic tree was generated using RaxmL v8.0.0 (Stamatakis 2014) based on the 120 bacterial single‐copy conserved genes from 101 draft or complete genomes from the *Anabaena*/*Dolichospermum*/*Aphanizomenon* (ADA) species complex (Tables S2 and S3) identified and aligned using GTDB‐tk v0.3.2 (Chaumeil et al. 2020). The phylogenomic tree was visualised and annotated using iTOL v6 (Letunic and Bork 2021). Average nucleotide identity (ANI) analysis was performed using Fast‐ANI v0.1.2 (Jain et al. 2018), which was then used to identify species in the ADA clade, as previously described (Dreher, Davis, and Mueller 2021). The distribution of the MAA biosynthetic gene clusters in public draft and complete genomes was identified using cblaster (Gilchrist et al. 2021). The gene synteny of the MAA biosynthetic gene cluster from the strains *Dolichospermum* sp. UHCC 0684 and *Dolichospermum* sp. UHCC 0260 was performed using Clinker (Gilchrist and Chooi 2021).

### 2.7 MAA Extraction

The biomass stored at −20°C was freeze‐dried at 0.0650 mbar using Christ LCS Plus Beta 2–8 LCS Plus Freeze Dryer (Martin Christ Gefriertrocknungsanlagen GmbH, Osterode am Harz, Germany) for 72 h. MAA extraction was carried out using 5 mg of freeze‐dried scum samples. Freeze‐dried biomass was placed in 2 mL plastic cryotubes with 1 mL of 100% methanol (Waters, LC/MS‐ grade), and 0.55 mm Glass Micro Beads (Scientific Industries, Bohemia, NY, USA) measured using filling a 200 μL plastic pipette tip. The material was homogenised at room temperature using a FastPrep‐24 cell disrupter (MP Biomedicals, Santa Ana, CA, USA) three times for 20 s at a speed of 6.5 m·s^−1^. The resulting cell debris was pelleted by centrifugation at 16,000×g for 5 min at room temperature. The supernatants were then transferred to 4 mL amber glass vials and stored at −20°C until further analysis. 300 μL of the methanol extract was mixed with 100% acetonitrile (VWR Chemicals, Darmstadt, Germany) (v:v 1:3) to efficiently precipitate protein residues from the sample, and the suspension was filtered through a 0.2 μm PTFE syringe filter (VWR International, Radnor, PA, USA).

### 2.8 LC–MS Analyses

MAAs were detected using an Acquity ultra‐performance liquid chromatography (UPLC) H‐Class PLUS Bio System (Waters, Manchester, UK) with Acquity Premier PDA detector (Waters Corp., Milford, MA, USA) based on their retention times and absorbance at 330 nm. Samples (MeOH‐ACN) were run using an Acquity UPLC BEH amide Column (100 × 2.1 mm, 1.7 μm, 130 Å, Waters, Manchester, UK) for separation. The sample injection volume was 0.1 μL and the column temperature was 40°C. Solvents A (0.1% HCOOH in milliQ water) and B (acetonitrile +0.1% HCOOH) were used with a flow rate of 0.3 mL min^−1^ from 85% of Solvent B for 7 min, then decreasing the percentage to 65% of B for 2.1 min, and then increasing back to 85% of B for the rest of the 15 min runtime.

Bloom samples from the year 2021 were run with Kinetex HILIC LC‐column (50 × 2.1 mm, 1.7 μm, 100 Å, Phenomenex) in UPLC‐PDA‐QTOF Acquity I‐Class UPLC‐Synapt G2‐Si (Waters Corp., Milford, MA) system. Bloom samples from the year 2022 were run with ACQUITY UPLC BEH Amide LC‐column (100 × 2.1 mm, 1.7 μm, 130 Å, Waters Corp., Milford, MA, USA) in Acquity Premier UPLC‐PDA‐QDA (Waters Corp., Milford, MA, USA) system. The solvents used in both systems were 0.2% ammonium formate in milliQ water (Solvent A) and acetonitrile (Solvent B) and 0.3 mL·min^−1^ flow rate and 40°C column oven temperature. The initial condition of the gradient for 2021 samples was 95% B and 5% A. B reached linearly 60% in 6.0 min and stayed in 60% until 8.0 min and increased linearly to 95% B by the time 8.5 min and stayed until 12.0 min. The initial condition of the gradient for 2022 samples was 90% B and 10% A. The B reached linearly 60% in 9.0 min and stayed in 60% until 10.0 min and increased linearly to 95% B by the time 10.1 and stayed until 16.0 min. Methanol‐extracted samples were diluted with acetonitrile (50 μL sample + 150 μL acetonitrile) and filtered with 0.2 μm PTFE syringe filters. Injection volume was 1 μL (2021) and 2 μL (2022). MAA peaks were integrated from 328 to 332 nm trace and Chlorophyll A peaks 654–656 nm trace.

The compounds detected from *Dolichospermum* spp. UHCC 0684 and UHCC 0260 in the UPLC analysis of the ACN‐diluted methanol extracts were confirmed as MAAs using a high‐resolution UPLC‐QTOF Acquity I‐Class UPLC‐Synapt G2‐Si (Waters Corp., Milford, MA, USA) system. Samples were analysed using ACQUITY UPLC BEH Amide Column (100 × 2.1 mm, 1.7 μm, 130 Å, Waters Corp., Milford, MA). The solvents used were 0.2% ammonium formate (Solvent A) and acetonitrile (Solvent B), 0.3 mL·min^−1^ flow rate and 40°C column oven temperature. The initial condition of the gradient was 90% B and 10% A. B reached linearly 60% in 9.0 min and stayed in 60% until 10.0 min and increased linearly to 95% B by the time 10.1 and stayed until 16.0 min. After electrospray ionisation samples were detected at positive polarity Resolution Mode at the mass range of *m/z* 50–2000. Ramp High Energy from 20.0 to 50.0 V and low energy at 10 V were the collision energy levels. Capillary voltage of 2.5 kV, sampling cone set to 20 V, with 120°C source temperature, 600°C of desolvation temperature, cone gas flow 50.0 L·h^−1^, desolvation gas flow 1000.0 L·h^−1^, nebulizer gas flow value 6.0 bar and collision energy 10 eV. QTOF was calibrated using sodium formate and Ultramark 1621. Leucine Enkephalin was used at 10‐s intervals as a lock mass reference compound. In MS/MS mode Trap Collision Energy Ramp proceeded from 20.0 to 40.0 eV.

### 2.9 Estimation of Porphyra‐334 Quantity in Cyanobacterial Strains

Freeze‐dried cells of cyanobacteria (10 mg) were extracted in 1 mL of methanol. 400 μL of extract was evaporated, dissolved in 200 μL of acetonitrile:methanol (75:25) solution, and filtered (0.2 μm). Injection volume was 1 or 2 μL. Xbridge PREMIER BEH Amide LC‐column (150 × 4.6 mm, 2.5 μm, Waters Corp., Milford, MA) in Acquity Premier UPLC‐PDA‐QDA‐FM (Waters Corp., Milford, MA) system was used. Isocratic elution was 25% of 0.2% ammonium formate and 75% acetonitrile, 1 mL·min^−1^ flow rate and 40°C column oven temperature. Porhyra‐334 fractions were collected, pooled, evaporated, and dissolved in 1 mL of water. UV–VIS spectrum was measured with a spectrophotometer (UV‐1800 Shimadzu, Kyoto, Japan) from 250 to 450 nm in a cuvette of 1 cm path length. Molar absorption coefficient of 42,300 M^−1^·cm^−1^ (La Barre and Kornprobst 2014) was used for concentration calculations.

### 2.10 Statistical Analysis

A Kruskal–Wallis (Hollander and Wolfe 1973) tests with post hoc Dunn's test (Dunn 1964) were performed to evaluate if the MAAs content between different locations (Helsinki and Turku) and years (2021 and 2022) differed statistically (*p* < 0.05).

## 3 Results

### 3.1 Detection of MAAs From Surface Scum Samples

**FIGURE 1:**
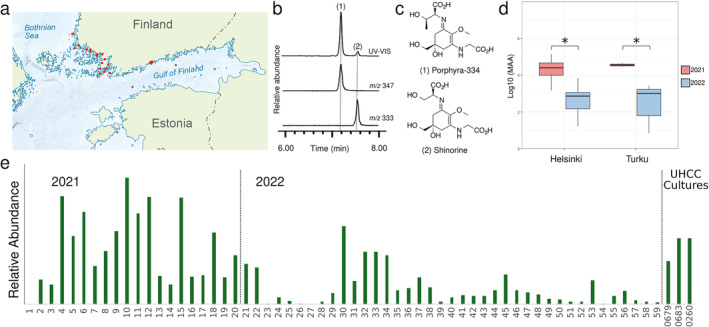
Detection of MAAs from samples of surface scums collected in 2021 and 2022. (a) Sampling locations marked by red dots. (b) Example HR‐LCMS and UV–vis spectra chromatograms with peaks 1 and 2 corresponding to porphyra‐334 and shinorine. (c) Chemical structures of porphyra‐334 and shinorine. (d) Boxplot of Log10‐transformed total relative amounts of MAAs in environmental samples collected from Helsinki and Turku during 2021 and 2022. Significant differences at *p* = 0.05 (*) were observed between years but not between areas. (e) Relative total MAA abundance of 59 surface scum samples collected from the southern coast of the Baltic Sea in 2021 and 2022.

We detected the presence of porphyra‐334 and shinorine in 54 of the 59 surface scum samples of cyanobacteria collected during the summers of 2021 and 2022 (Figure 1 and Table S1). Surface bloom samples collected in 2021 (*n* = 20) showed a 10‐fold higher content of MAA content in comparison to 2022 (*n* = 39) (Figure 1 and Table S1). The highest total MAA accumulation was detected in early July to mid‐August in 2021, which came from bloom samples no. 4, 10, 12, and 15, taken from Laajalahti, Espoo, Matinkylä beach, Laajasalo beach, and Munkkiniemi beach, respectively (Figure 1 and Table S1). These samples primarily contained porphyra‐334 and shinorine in varying ratios (Figures 1 and S8). In these MAA‐rich samples, shinorine was predominant except in sample no. 15, where the porphyra‐334 peak area was 15 times larger than that of shinorine on the MS ES+ chromatograms (Figure S8). The 2022 bloom samples accumulated comparable MAA amounts to our UHCC strains 0679, 0683, and 0260 that are maintained under constant low light. The Wallis test with post hoc Dunn's test was carried out with the aim to reveal significative differences in the MAAs content between areas and years. The test revealed significant differences (Figure 1 and Table S9) between the MAAs content of year 2021 and 2022 but not between areas (Helsinki and Turku). The results show an overall decrease in MAAs content in 2022 compared with 2021. Interestingly, the data also reveal a consistent trend of homogeneity in MAAs content between Helsinki and Turku, despite the nearly 200 km of coastline separating the two regions.

### 3.2 Analysis of MAA Biosynthetic Gene in Surface Scum Sample

**FIGURE 2:**
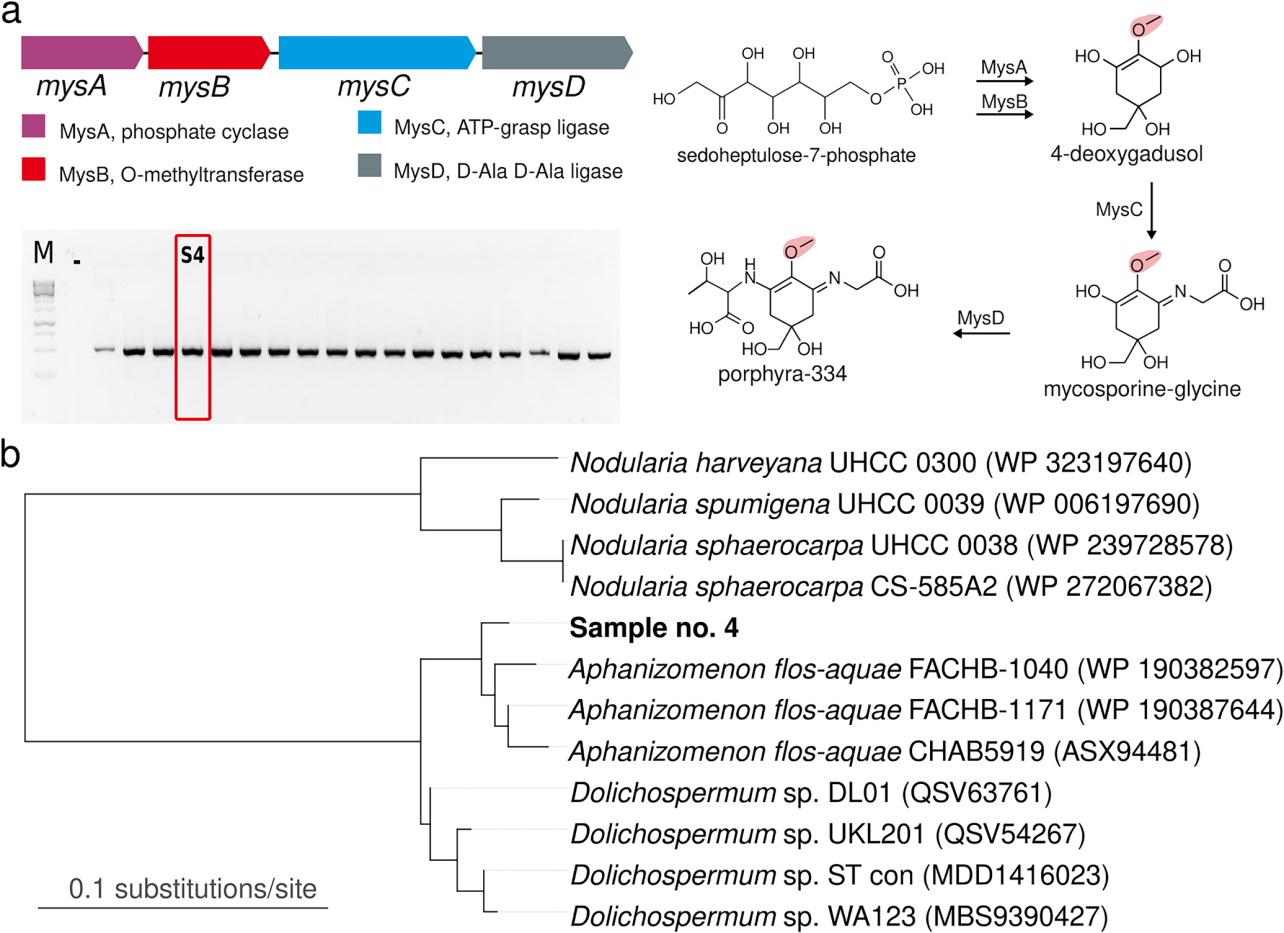
(a) Detection of the *mysB* gene encoding an O‐methyltransferase as part of the MAA biosynthetic gene cluster from sample number no. 4 (complete gel image including all samples and positive controls provided in Figure S2) and the established porpyhra‐334 biosynthesis based on the pathway presented by Balskus and Walsh 2010. (b) Phylogenetic inference of MAA‐producing organism from sample no. 4 based on the MysB amino acid sequence.

The scum samples frequently contained members of the filamentous diazotrophic and bloom‐forming ADA species complex and *N. spumigena* towards the late summer (Table S1 and Figure S2). We developed an endpoint PCR method based on the *mysB* biosynthetic gene to allow the culture‐independent discrimination of MAA‐producing cyanobacteria directly from environmental samples (Figures 2 and S2). We extracted genomic DNA from the 59 samples of cyanobacterial collected during the summers of 2021 and 2022 and amplified 486‐bp region of the *mysB* biosynthetic gene by PCR (Figure S2). We detected the presence of the *mysB* biosynthetic gene in all 59 surface scum samples, even from the seven bloom samples for which neither porphyra‐334 nor shinorine were detected by HR‐LCMS analysis (Figure S2 and Table S1). We selected sample no. 4 as the representative sample as it was one of the highest MAA containing one in the sample set and sequenced its *mysB* gene (Figure 2 and Table S1). The 157‐amino‐acid fragment of MysB from sample no. 4 shared 97.53% sequence identity with the MysB protein from the genome of *A. flos‐aquae* FACHB‐1171 in BlastP searches against the non‐redundant database at NCBI (data not shown). We constructed a maximum‐likelihood phylogenetic tree based on public MysB (Figures 2 and S3). The MysB fragment from sample no. 4 formed a group with MysB proteins from members of the *Aphanizomenon* and *Dolichospermum* genera (Figures 2 and S3).

### 3.3 Distribution of MAA Biosynthetic Gene Clusters in ADA Species Complex

**FIGURE 3:**
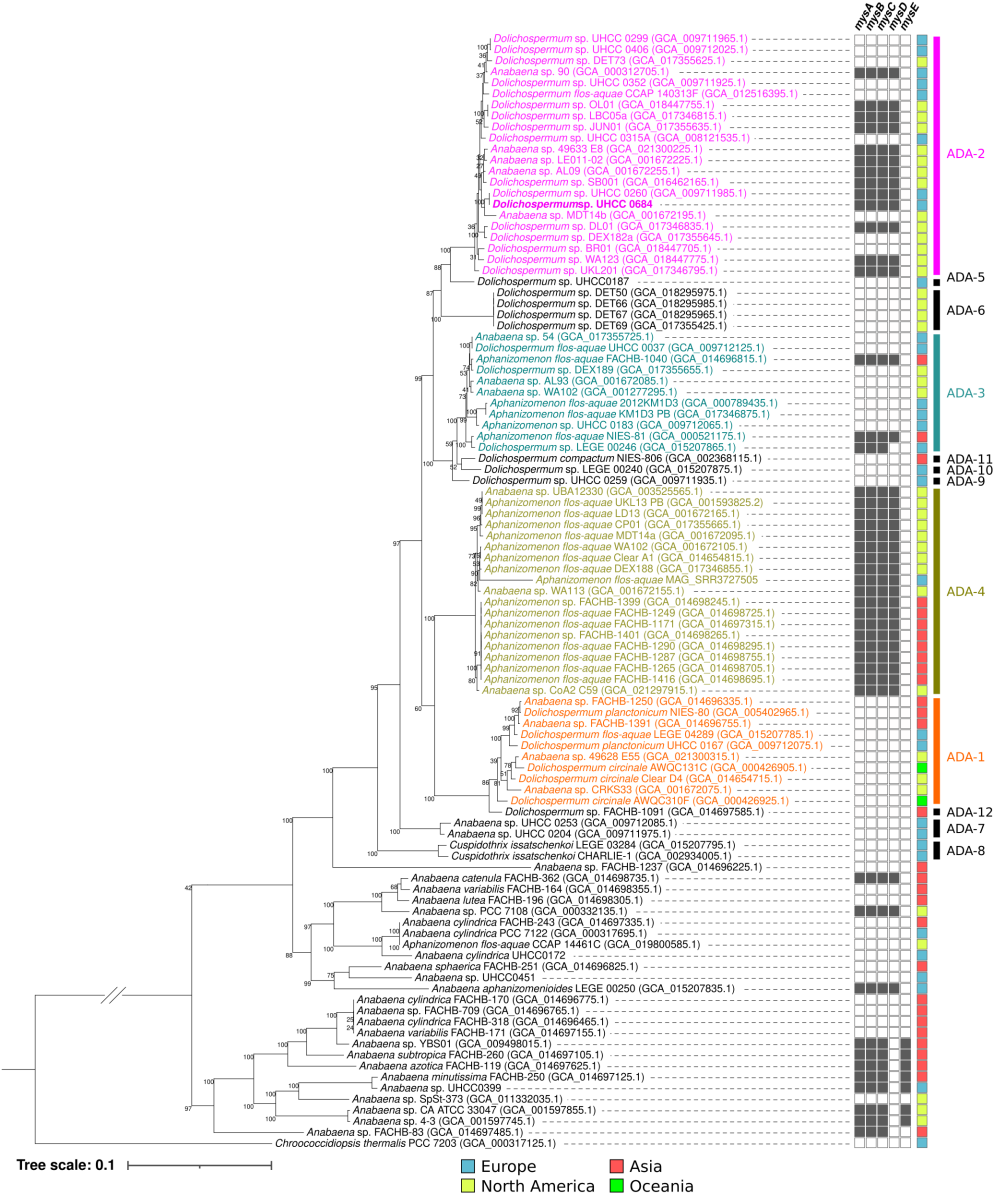
Phylogenomic tree illustrating the distribution of MAA biosynthetic pathways (*mysA*‐*mysE*, in black boxes) in genomes of members of the ADA species complex. The phylogenomic tree is based on 120 bacterial single copy conserved genes from 101 draft or complete genomes using ANI cut‐off values of 96% (Figure S4). The ADA clade represents a genus‐level complex, encompassing 12 species: From ADA‐1 to ADA‐12, identified with an ANI analysis following the practice proposed by Dreher, Davis, and Mueller (2021) and Dreher et al. (2021) (Figure S4). *Dolichospermum* sp. UHCC 0684 is highlighted in bold.

We analysed the prevalence of the MAA biosynthetic pathway in the genomes of the closely related ADA genera of toxic and bloom‐forming cyanobacterial (Figure 3 and Table S2). The strains included in this analysis originate from a range of geographical origins in Europe, Asia, and North America (Figure 3 and Table S2). The ANI analysis values calculated from the 101 analysed genomes identified 12 distinct species within the ADA clade that we nominally labelled ADA‐1 through ADA‐12 (Figures 3 and S4). We identified 47 complete or partial MAA biosynthetic gene clusters from the 101 analysed ADA genomes (Figure 3 and Table S3). The distribution of MAA biosynthetic enzymes mapped to the phylogenomic tree suggested that 48% of the species in the ADA clade had the biosynthetic genes required to produce MAAs (Figure 3 and Table S3). MAA biosynthetic enzymes were especially concentrated in strains from Asia and North America within ADA‐2, comprised of *Dolichospermum* strains, and ADA‐4, with *Aphanizomenon* strains (Figure 3). With MAA biosynthetic pathways present in all its strains, ADA‐4 alone represented 52.8% of the MAA biosynthetic pathways in all 12 identified ADA species (Figure 3 and Table S3).

### 3.4 Detection of MAAs From Strains of *Dolichospermum*

**FIGURE 4:**
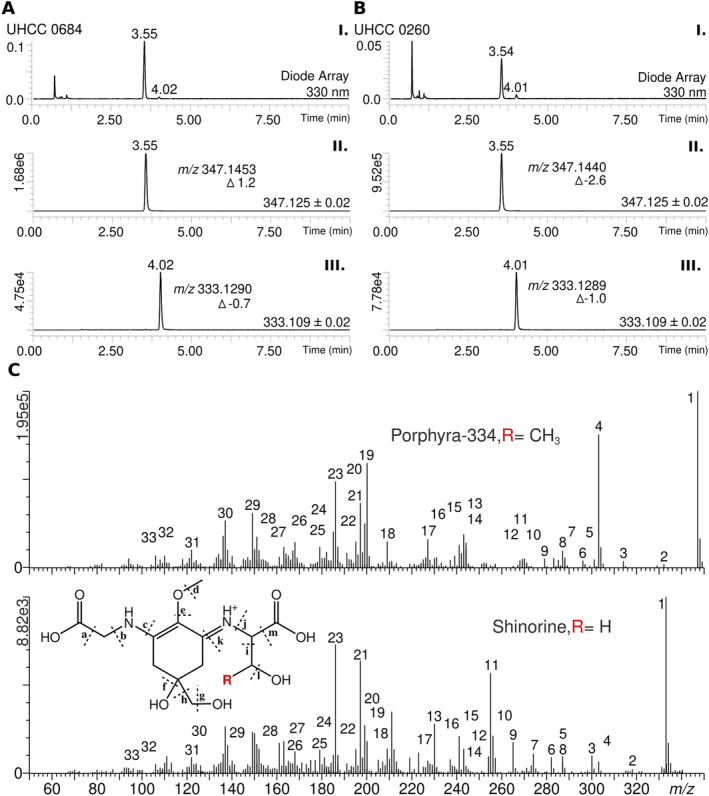
LC/MS‐QTOF analysis of MAA detected from two *Dolichospermum* strains UHCC 0684 (a) and UHCC 0260 (b) as follows: I. UV chromatogram 330 nm with retention times of porphyra‐334 (3.55 min) and shinorine (4.02 min) presented above peaks. II. Extracted ion (*m/z* 347.145 ± 0.02) chromatogram (EIC) showing peak of porphyra‐334 (*m/z* 347.1453). III. EIC (*m/z* 333.129 ± 0.02) showing peak of shinorine (*m/z* 333.1290). I. UV chromatogram 330 nm with retention times of porphyra‐334 (3.54 min) and shinorine (4.01 min) presented above peaks. II. EIC showing peak of porphyra‐334 (*m/z* 347.1440). III. EIC showing peak of shinorine (*m/z* 333.1289). Δ marks error in parts per million (ppm). MS^E^ (E: Elevated collision energy) spectra of the porphyra‐334 peak at 3.54 min and shinorine peak at 4.01 min from *Dolichospermum* sp. UHCC 0260 (c). Annotation of the numbered fragment ions are presented in Table S6.

We screened 36 *Dolichospermum* strains from the UHCC of cyanobacteria for the production of MAAs using HR‐LCMS analysis (Table S4). We detected porphya‐334 and shinorine in five of the 36 examined *Dolichospermum* strains isolated from surface water from the Gulf of Finland (Figure 4 and Tables S4–S6). *Dolichospermum* spp. UHCC 0684 and UHCC 0260 were observed to produce relatively higher amounts of porphya‐334 and shinorine (Table S5) leading to their selection for further analysis of these samples in HR‐LCMS analysis to confirm the identity of the compounds (Figure 4 and Tables S4–S6). The HR‐LCMS analysis supports the assignment of the compounds detected in the preliminary screening as porphyra‐334 and shinorine; two ions with the masses of *m/z* 347.1 and *m/z* 333.1 were observed, corresponding to typical mass‐to‐charge ratios of protonated porphyra‐334 and shinorine, respectively (Tables S7 and S8). The assignment of the most abundant compound as porphyra‐334 is further reinforced by the fragmentation patterns of observed ion with *m/z* 347.1453 (Figure 4 and Table S6) from *Dolichospermum* sp. UHCC 0684, the sample producing the highest relative amount of porphyra‐334 (Figure 4 and Table S5). Many of the putative fragments and their *m/z* ratios are typical to porphyra‐334 extracted from cyanobacterial samples (Figure 4 and Table S6). However, while porphyra‐334 and shinorine were present in both strains, porphyra‐334 was more abundant in each case (Table S5). The estimated quantity of porphyra‐334 was 7.4 mg·g^−1^ DW in *Dolichospermum* sp. UHCC 0684, considerably higher than the 2.6 mg·g^−1^ DW detected in the strain *Dolichospermum* sp. UHCC 0260 (Figures S5 and S6). We obtained draft genomes *Dolichospermum* spp. UHCC 0684 and UHCC 0260 and identified the MAA biosynthetic gene cluster (Figures 3 and S7). The *mysA*–*mysD* genes and promoter region from *Dolichospermum* sp. UHCC 0684 and *Dolichospermum* sp. UHCC 0260 were 100% identical at the nucleotide level (Figure S7).

## 4 Discussion

We detected porphyra‐334 and shinorine in 52 of the 59 samples of surface scum samples collected from the coastal regions of the Baltic Sea in the summers of 2021 and 2022 (Figures 1 and S1 and S2 and Table S1). The highest relative abundance of MAAs came from the samples collected during the summer of 2021 (Figure 1). The results obtained from the statistical analysis performed suggest that common environmental factors or broader climatic patterns may be influencing the MAA production in both areas, as previously suggested for cyanobacterial blooms (Tromas et al. 2017). The weather conditions in 2021 may have been more favourable to produce MAAs than in 2022 (Figure 1). Nutrient availability could play a role in MAA abundance, for example, phosphorous levels are expected to be declining in this area of the Baltic Sea (Müller‐Karulis et al. 2024). Mineral phosphorous concentration in The Baltic Sea were reported to have remained low throughout the year in 2022 and ranged from 0.001 to 0.074 mg P/dm^3^, showing a decrease compared with previous years (Siniakova, Krylova, and Bronnikova 2024). The authors specify that the content of biogenic elements in the waters of the Gulf of Finland was lower than in 2020 and 2021, with median total phosphorous concentration at the oligotrophic level (Siniakova, Krylova, and Bronnikova 2024). We also cannot exclude the possibility that the observed differences are due to distinct sample volumes collected (1–2 L), despite attempts to minimise discrepancies by extracting from the same amount of dry weight. However, in both 2021 and 2022, samples with the highest total MAA content were collected in August and in the Helsinki area (Figure 1 and Table S1). Further investigation could explore whether temperature variations, UV radiation levels, or nutrient availability might be contributing to the observed temporal and regional trends.

MAA biosynthetic gene clusters are reported to be subject to rapid evolutionary processes resulting in highly plastic biosynthetic pathways, which generate the chemical diversity of MAAs (Arsın et al. 2023). Members of the ADA species complex and *N. spumigena* are the most frequently reported scum forming cyanobacteria in the Baltic Sea (Sivonen, Poon, and Codd 1989; Sivonen et al. 1990; Teikari et al. 2019). Members of both are reported to produce MAAs in culture (Sinha et al. 2003; Jacinavicius et al. 2021; Zhang et al. 2022). We developed an endpoint PCR method based on the *mysB* biosynthetic gene to allow the differentiation of MAA‐producing cyanobacteria from the ADA clade directly from environmental samples without the need to isolate cultures (Figures 2 and S2). We extracted genomic DNA from the 59 bloom samples containing cyanobacterial collected during the summers of 2021 and 2022 and amplified 486‐bp region of the *mysB* biosynthetic gene by PCR (Figure S2). We detected the presence of the *mysB* biosynthetic gene in all 59 surface scum samples, even from the bloom samples for which neither porphyra‐334 nor shinorine were detected by HR‐LCMS analysis (Figure S2 and Table S1), and sequenced the fragment from a representative sample (Figure 2). Phylogenetic analysis of the 157‐amino‐acid fragment of MysB from sample no. 4 shared 97.53% sequence identity with the MysB protein from the genome of *A. flos‐aquae* FACHB‐1171 (Figure 2) and was placed in a clade alongside other bloom‐forming cyanobacteria from the ADA species complex (Figure  2).

The ADA species complex genera *Dolichospermum* and *Aphanizomenon* dominate a large portion of the annual cyanobacterial blooms in the Baltic Sea during the summer (Sivonen, Poon, and Codd 1989; Sivonen et al. 1990; Teikari et al. 2019). The surface blooms forming on the topmost layers of the water column are exposed to intense solar ultraviolet radiation (Torres et al. 2006). These blooms are exposed to intense solar ultraviolet radiation (Torres et al. 2006), so it is likely that these species produce natural microbial sunscreens to protect themselves. MAAs have been reported from cyanobacteria in the ADA clade (Jacinavicius et al. 2021; Zhang et al. 2022) and from *A. flos‐aquae* harvested as ingredients of dietary supplement capsules (Torres et al. 2006; Righi et al. 2016). However, overall MAAs from bloom‐forming cyanobacterial species are sparsely reported (Liu, Häder, and Sommaruga 2004; Sommaruga, Chen, and Liu 2009).

The ADA species complex has numerous complete or draft genomes available (Dreher et al. 2021), and recent publications have brought insight into their metabolism (Driscoll et al. 2018) and toxin biosynthetic pathways (Österholm et al. 2020). We conducted phylogenomic analysis to determine the frequency of the MAA biosynthetic pathways in members of the ADA clade by mapping the distribution of MAA biosynthetic enzymes was mapped to a phylogenomic tree based on 120 bacterial single‐copy conserved genes from 101 draft or complete genomes from the ADA species complex (Figure 3). The results revealed that the occurrence of MAA biosynthetic gene clusters in this species complex is concentrated on ADA‐2 and ADA‐4, which contain strains from *Dolichospermum*, and *Aphanizomenon* genera, respectively (Figure 3). Apart from one exception of *Dolichospermum* sp. LEGE 00246 in ADA‐3, genes *mysABCD* were present in all ADA strains possessing this biosynthetic gene cluster, indicating this structure of the pathway as conserved among the species complex (Figure 3). This finding is supported by existing reports on the conservation of *mysABC* genes among cyanobacteria (Balskus and Walsh 2010; Gao and Garcia‐Pichel 2011b). All ADA‐4 strains have the biosynthetic pathway to produce MAAs based on phylogenomic analysis (Figure 3 and Table S3). It is widely reported that MAAs play an important role in protecting aquatic organisms against UV damage (Tartarotti and Sommaruga 2006). The sporadic distribution of the MAA biosynthetic pathway suggests that the different species of the ADA species complex may have different strategies for coping with consistent environmental stress.

We detected shinorine and porphyra‐334 in five examined *Dolichospermum* strains isolated from surface water from the Gulf of Finland with porphyra‐334 as the dominant variant in each case (Tables S4 and S5). We estimated that *Dolichospermum* sp. UHCC 0684 produced 7.4 mg·g^−1^ of porphyra‐334 in the absence of UV induction as the dominant MAA variant (Tables S7 and S8). Porphyra‐334 and shinorine were recently reported from *A. flos‐aquae* and *Aphanizomenon gracile*, with quantities ranging from 0.003 to 0.497 mg·g^−1^ of total MAAs (Zhang et al. 2022). This production level is typical to be found in cyanobacteria, as MAA concentrations reported from cyanobacteria rarely exceed the value of 1.0 mg g^−1^, unless production is additionally induced (Rastogi, Madamwar, and Incharoensakdi 2015; Hartmann et al. 2015; Inoue‐Sakamoto et al. 2018; Geraldes et al. 2020). However, exceptions have been observed, and UV‐induction often leads to increased production, as seen in the case of cyanobacteria genera such as *Nodularia*, *Nostoc*, *Pseudanabaena*, and *Sphaerospermopsis* (Geraldes et al. 2020; Boucar et al. 2021). We obtained draft genome sequences for the two high MAA‐producing *Dolichospermum* strains: UHCC 0684 and UHCC 0260, in order to investigate thepotential differences in their MAA biosynthetic pathways (Figures  4 and S5). Both strains possessed the same MAA biosynthetic pathway enzymes in the *mysABCD* organisation as well as the promoter region, all of which sharing 100% identity at protein and nucleotide level (Figure S7). Phylogenomic analysis also placed the two strains next to each other in the same clade, indicating a close relationship (Figure 3). Despite these similarities, UHCC 0684 is able to produce much higher levels of porphyra‐334 compared to UHCC 0260 (Table S7 and Figure S5). Potential explanations for the differences in MAA production levels may be due to differences in complex regulatory pathways (Gao and Garcia‐Pichel 2011a, 2011b; Oren and Gunde‐Cimerman 2007). Overall, the abundance of MAAs in different producing organisms fluctuates, in lichen they vary between approximately 10 and 20 mg·g^−1^ DW (Chollet‐Krugler et al. 2019), and the concentrations reported from red algae typically range from 2 to 10 mg·g^−1^ DW (Hartmann et al. 2015; Lawrence, Long, and Young 2018; Sun et al. 2020; Figueroa 2021).

Our findings suggest that bloom forming cyanobacteria belonging to ADA complex species from the Baltic Sea might be good source of MAAs (Figures 2 and 3). Especially high MAA production was observed in *Dolichospermum* sp. UHCC 0684 and *Dolichospermum* sp. UHCC 0260 which were previously isolated from Baltic Sea bloom samples (Figure 4 and Tables S7 and S8). This is highly likely to be due to extensive UV exposure of cyanobacteria that form surface blooms and scums (Medrano et al. 2016). Alongside the effective UV protection, MAAs have also been shown to be multipurpose stress protectants with their abilities to scavenge harmful radicals, act as compatible solutes against salt stress, and alleviate the effects of desiccation (Oren and Gunde‐Cimerman 2007). In addition to their multifunctionality in microorganisms, plenty of studies also reported on their general stability and non‐toxic nature alongside additional useful properties such as anti‐inflammatory, anti‐aging, and anti‐tumour activities that could be harnessed by pharmaceutical and cosmetic industries (Peng et al. 2023). The interest in MAAs as future sunscreen compounds has also grown in recent years due to the limitations of current cosmetic photoprotective products (Chrapusta et al. 2017; Wnuk et al. 2022; Verma et al. 2024). UV‐A and UV‐B both harm the skin, but many commercial products only protect against UV‐B (Mancuso et al. 2017). Additionally, some sunscreen ingredients can release harmful substances when exposed to UV‐A radiation, causing damage to the skin (Millington, Osmond, and McCall 2014). These ingredients may also penetrate too deeply or irritate the skin, and they may accumulate in aquatic organisms and harm the food chain (Singh et al. 2021). Understanding more about the production and availability of MAAs can help in creating safe and eco‐friendly UV filters (Gao and Garcia‐Pichel 2011a, 2011b). Examples of these are Helioguard365, a commercially available sunscreen product containing porphyra‐334 and shinorine with anti‐aging and UV‐A protective properties (Schmid et al. 2003), and Helionori, another *Porphyra*‐based natural photoprotective product containing a combination of porphyra‐334, shinorine and palythine (Lawrence, Long, and Young 2018; Singh et al. 2021). However, the low abundance of MAAs and the difficulties in finding good producers in laboratory conditions pose a challenge for researching industrial applications of these compounds (Geraldes et al. 2020). The knowledge gained from studying MAAs in their natural environment is essential not only for understanding their ecological roles but also to investigate the potential of cyanobacterial blooms as natural reservoir. The outcomes of this study could provide insights into the evolutionary relationships and ecological functions of these organisms, contributing to the development a model that considers the weather conditions of the Baltic Sea area to enhance the ability to identify blooms with high MAAs content. Further analyses and experiments will be necessary to confirm and expand upon these initial findings.

## 5 Conclusions

This study investigated the presence of MAAs in surface bloom‐forming cyanobacteria. Our findings demonstrate the widespread occurrence of MAA biosynthetic pathways, with the production of shinorine and porphyra‐334 being particularly prevalent in species belonging to the ADA complex species. These cyanobacteria could be viewed as potential natural reservoirs of MAAs which could be harnessed for future biotechnological applications. Following up on this, we identified two prolific MAA producing *Dolichospermum* sp. previously isolated from Baltic Sea blooms and maintained in UHCC. Notably, MAA production in *Dolichospermum* sp. UHCC 0684 appears to be highly up‐regulated, highlighting the need for further investigation into the regulatory pathways that control MAA biosynthesis. Finally, our results provide valuable insights for future research. The high prevalence of MAA biosynthetic pathways in bloom‐forming cyanobacteria suggests that MAAs may play a role in the formation and persistence of surface blooms and scums, warranting further exploration of their ecological function.

## Author Contributions

**Inkeri Vuori:** writing – original draft, methodology, validation, visualization, writing – review and editing, formal analysis, data curation. **Greta Gaiani:** methodology, validation, writing – original draft, visualization, writing – review and editing, formal analysis, data curation, supervision. **Sıla Arsın:** writing – original draft, funding acquisition, methodology, validation, visualization, writing – review and editing, formal analysis, software, data curation, project administration, supervision. **Endrews Delbaje:** writing – original draft, methodology, validation, visualization, writing – review and editing, software, formal analysis, data curation. **Julia Järn:** formal analysis, methodology, validation, data curation. **Robert Snårbacka:** software, formal analysis, data curation, methodology, validation. **Annaël Couëdelo:** methodology, validation, software, formal analysis, data curation. **Gayathri Murukesan:** writing – original draft, methodology, validation, visualization, writing – review and editing, formal analysis, software, data curation, supervision. **Matti Wahlsten:** writing – original draft, methodology, validation, visualization, writing – review and editing, software, formal analysis, data curation. **Jouni Jokela:** data curation, software, formal analysis, writing – review and editing, visualization, validation, methodology, investigation, writing – original draft. **Tânia Keiko Shishido:** funding acquisition, writing – original draft, methodology, validation, visualization, writing – review and editing, software, formal analysis, data curation, supervision. **David P. Fewer:** conceptualization, investigation, funding acquisition, writing – original draft, writing – review and editing, visualization, validation, software, formal analysis, project administration, methodology, data curation, supervision, resources.

## Conflicts of Interest

The authors declare no conflicts of interest.

## Supporting information


**Data S1** Supporting Information.

## Data Availability

The data that support the findings of this study are openly available in GenBank, reference numbers GCA_034931625.1, GCA_034931465.1, GCA_034931405.1, and GCA_009712.
