# Supplementary material for: Direct Evidence of Microbial Sunscreen Production by Scum‐Forming Cyanobacteria in the Baltic Sea
Source: Environ Microbiol Rep. 2025 Jan 16;17(1):e70056. doi: 10.1111/1758-2229.70056 (PMC11738650; doi:10.1111/1758-2229.70056)
Supplement: Supplementary file 1 — Data S1 Supporting Information. [file EMI4-17-e70056-s001.pdf]

Table S1. Bloom samples collected in 2021 and 2022. Sample number, sampling location, total relative abundance of MAAs, Chl A levels, dry weight per methanol extraction and genera observed are reported in the table.

Bloom Sample No.	Location	Date	Coordinates (DMS)	LCMS ID	MAA (Total)	Chl a (654-656 nm)	MAA/Chl a	Dry weight (mg)	Genera observed
1	Öbyviken,Tammisaari	21.6.2021	59°55'28.2"N 23°09'32.7"E	G111	0	697	0	5	cf *Limnoraphis robusta*
2	Munkkiniemi, Helsinki	30.6.2021	60°12'01.1"N 24°51'26.3"E	G112	724	1361	0,5319618	5	cf *Aphanizomenon flos-aquae*, cf *Dolichospermum*
3	Koivusaari, Helsinki	1.7.2021	60°09'42.6"N 24°51'16.5"E	G113	1468	3442	0,4264962	5	cf *Aphanizomenon flos-aquae*, cd *Dolichospermum*
4	Laajalahti, Espoo	8.7.2021	60°12'21.0"N 24°50'16.0"E	G129	140691	61415	2,2908247	5	cf *Aphanizomenon flos-aquae*, cf *Dolichospermum*
5	Eningsholmintie, Kustavi	2.8.2021	60°30'12.7"N 21°17'10.4"E	G153	28441	19606	1,4506274	5	cf *Dolichospermum*, cf *Aphanizomenon flos-aquae*
6	Lyökki, Uusikaupunki	2.8.2021	60°51'36.2"N 21°11'22.2"E	G147	35822	18199	1,9683499	5	cf *Nodularia spumigena*, cf *Dolichospermum*, cf *Aphanizomenon flos-aquae*
7	Taivassalo	2.8.2021	60°33'44.6"N 21°32'09.7"E	G148	22483	27608	0,8143654	5	cf *Nodularia spumigena*, cf *Dolichospermum*, cf *Aphanizomenon flos-aquae*
8	Soukka, Espoo	3.8.2021	60°08'11.0"N 24°39'06.3"E	G149	25399	22443	1,1317114	5	cf *Dolichospermum*, cf *Aphanizomenon flos-aquae*
9	Westend beach, Espoo	9.8.2021	60°10'07.3"N 24°48'37.2"E	G154	47045	30098	1,5630607	5	cf *Dolichospermum*, cf *Aphanizomenon flos-aquae*
10	Matinkylä beach	9.8.2021	60°09'03.0"N 24°45'22.4"E	G155	50955	18973	2,6856586	5	cf *Dolichospermum*, cf *Aphanizomenon flos-aquae*
11	Aurinkolahti beach	9.8.2021	60°12'07.4"N 25°09'35.2"E	G156	26986	13936	1,9364237	5	cf *Dolichospermum*, cf *Nodularia spumigena*
12	Laajasalo beach	9.8.2021	60°10'37.6"N 25°03'50.4"E	G157	25075	11018	2,2758214	5	cf *Dolichospermum*, cf *Aphanizomenon flos-aquae*, cf *Nodularia spumigena*
13	Suomenlinna west	12.8.2021	60°08'42.3"N 24°58'47.6"E	G158	11849	19722	0,6008011	5	cf *Dolichospermum*, cf *Aphanizomenon flos-aquae*, cf *Nodularia spumigena*
14	Suomenlinna east	12.8.2021	60°08'28.4"N 24°59'13.8"E	G159	12964	30397	0,4264895	5	cf *Dolichospermum*, cf *Nodularia spumigena*
15	Munkkiniemi beach	12.8.2021	60°09'15.0"N 24°56'37.2"E	G160	46327	20365	2,2748343	5	cf *Dolichospermum*
16	Kaivopuiston ranta	12.8.2021	60°09'15.0"N 24°56'37.2"E	G161	9907	17047	0,581158	5	cf *Dolichospermum*, cf *Aphanizomenon flos-aquae*, cf *Nodularia spumigena*
17	Norrbyviken, Parainen	24.8.2021	60°18'48.4"N 22°17'51.1"E	G165	40001	65240	0,6131361	5	cf *Nodularia spumigena*, cf *Aphanizomenon flos-aquae*
18	Naantali marina	24.8.2021	60°28'11.1"N 22°00'56.7"E	G166	47191	30936	1,5254396	5	cf *Nodularia spumigena*, cf *Aphanizomenon flos-aquae*, cf *Dolichospermum*
19	Kvarnholm, Parainen	24.8.2021	60°19'21.8"N 22°20'31.1"E	G167	40099	70723	0,5669867	5	cf *Nodularia spumigena*

20	Hiirsalmi, Piikkiö, Turku	24.8.2021	60°24'07.7"N 22°29'18.0"E	G168	35691	34209	1,0433219	5	cf *Nodularia spumigena*, cf *Aphanizomenon flos-aquae*, cf *Dolichospermum*
21	Rajasaari	5.7.2022	60°10'57.7"N 24°54'37.0"E	GG01	1522	1785	0,8526611	5,4	cf *Nodularia*, cf *Anabaena,* cf *Aphanizomenon*
22	Rajasaari	8.7.2022	60°10'50.3"N 24°54'29.9"E	GG02	2246	2873	0,7817612	5,5	cf *Nodularia*, cf *Anabaena,* cf *Aphanizomenon*
23	Munkkiniemi	11.7.2022	60°12'03.0"N 24°51'24.4"E	GG03	1	89	0,011236	4,5	cf *Anabaena,* cf *Dolichospermum,* cf *Apanizomenon*
24	Rajasaari	11.7.2022	60°10'52.1"N 24°54'33.1"E	GG04	527	3590	0,1467967	5	cf *Nodularia*, cf *Anabaena,* cf *Aphanizomenon*
25	Puotila	11.7.2022	60°12'22.0"N 25°06'09.3"E	GG05	120	1847	0,0649702	5,1	cf *Nodularia*, cf *Anabaena,* cf *Aphanizomenon*
26	Puotila beach	11.7.2022	60°12'30.7"N 25°06'32.7"E	GG06	16	2016	0,0079365	5	cf *Anabaena,* cf *Dolichospermum,* cf *Aphanizomenon*
27	Munkkiniemi	11.7.2022	60°12'12.3"N 24°51'06.8"E	GG07	0	1378	0	4,8	cf *Anabaena,* cf *Dolichospermum,* cf *Aphanizomenon*
28	Klobben	18.7.2022	60°08'06.2"N 24°38'52.3"E	GG08	93	1479	0,0628803	5,1	cf *Anabaena,* cf *Dolichospermum,* cf *Aphanizomenon*
29	Munkkiniemi	19.7.2022	60°12'03.0"N 24°51'24.4"E	GG09	821	3488	0,2353784	5,2	cf *Anabaena,* cf *Dolichospermum,* cf *Aphanizomenon,* cf *Nodularia*
30	Rajasaari	28.7.2022	60°10'59.1"N 24°54'35.7"E	GG20	7027	4239	1,6577023	5,9	cf *Anabaena,* cf *Dolichospermum,* cf *Aphanizomenon,* cf *Nodularia*
31	Laajasalo beach	1.8.2022	60°10'34.9"N 25°03'48.6"E	GG22	737	1495	0,4929766	6,08	cf *Dolichospermum*, cf *Aphanizomenon, cf Nodularia*
32	Lapinlahti	2.8.2022	60°10'05.2"N 24°54'54.1"E	GG24	3909	3490	1,1200573	5,07	cf *Dolichospermum*, cf *Aphanizomenon, cf Nodularia*
33	Rajasaari	5.8.2022	60°10'57.6"N 24°54'37.5"E	GG35	1628	1454	1,1196699	5,3	cf *Dolichospermum*, cf *Aphanizomenon, cf Nodularia*
34	Pikisaari beach	9.8.2022	60°10'57.6"N 24°54'37.5"E	GG41	2021	1952	1,0353484	6	cf *Aphanizomenon*
35	Vesiläntie	9.8.2022	60°35'11.8"N 21°49'26.4"E	GG50	518	1717	0,301689	6,7	cf *Aphanizomenon*, cf *Nodularia,* cf *Anabena,* cf *Dolichospermum*
36	Kultaranta	9.8.2022	60°27'47.4"N 22°00'12.2"E	GG52	1906	5555	0,3431143	5,9	cf *Nodularia*, cf *Anabaena*, cf *Dolichospermum*
37	Laajasalo beach	10.8.2022	60°10'34.9"N 25°03'48.6"E	GG57	990	1719	0,5759162	5,5	cf *Aphanizomenon*, cf *Nodularia,* cf *Anabena,* cf *Dolichospermum*
38	Marjaniemi Beach	10.8.2022	60°11'54.7"N 25°04'34.8"E	GG62	714	1994	0,3580742	5,4	cf *Aphanizomenon*, cf *Nodularia,* cf *Anabena,* cf *Dolichospermum*
39	Rajasaari (Boat club)	10.8.2022	60°10'52.1"N 24°54'33.1"E	GG77	116	2226	0,0521114	5,7	cf *Aphanizomenon*, cf *Nodularia,* cf *Anabena,* cf *Dolichospermum*
40	Rajasaari	10.8.2022	60°10'57.7"N 24°54'37.4"E	GG78	515	3582	0,1437744	5,5	cf *Aphanizomenon*, cf *Nodularia,* cf *Anabena,* cf *Dolichospermum*
41	Rajasaari	10.8.2022	60°10'57.7"N 24°54'37.4"E	GG79	1146	5817	0,1970088	6,7	cf *Dolichospermum*, cf *Aphanizomenon, cf Nodularia*

42	Rajasaari (Boat club)	12.8.2022	60°10'52.1"N 24°54'29.3"E	GG80	1090	6150	0,1772358	6,5	cf *Dolichospermum*, cf *Aphanizomenon, cf Nodularia*
43	Rajasaari (Boat club)	12.8.2022	60°10'52.5"N 24°54'34.0"E	GG82	897	4922	0,182243	5,7	cf *Dolichospermum*, cf *Aphanizomenon, cf Nodularia*
44	Kirjalansaari, Parainen	16.8.2022	60°19'42.6"N 22°23'21.1"E	GG91	1035	3434	0,3013978	5,6	cf *Dolichospermum*, cf *Aphanizomenon, cf Nodularia*
45	Hovirinta marina, Kaarina	16.8.2022	60°23'43.7"N 22°22'19.6"E	GG96	2663	4222	0,6307437	5,5	cf *Nodularia*, cf *Dolichospermum*, cf *Aphanizomenon*
46	Harvaluodon uimaranta	16.8.2022	60°22'14.7"N 22°30'58.8"E	GG99	1008	3452	0,2920046	5,8	cf *Nodularia*, cf *Dolichospermum, cf Aphanizomenon,*
47	Katajanokka	18.8.2022	60°10'09.2"N 24°57'50.4"E	GG106	1149	4549	0,252583	5,4	cf *Aphanizomenon*, cf *Nodularia,* cf *Dolichospermum*
48	Katajanokka	18.8.2022	60°10'08.9"N 24°57'57.4"E	GG108	987	4815	0,2049844	5,5	cf *Aphanizomenon*, cf *Nodularia,* cf *Dolichospermum*
49	Ruoholahti	18.8.2022	60°09'42.5"N 24°54'49.6"E	GG111	288	2407	0,119651	5,5	cf *Dolichospermum*, cf *Aphanizomenon, cf Nodularia*
50	Otaniemi	18.8.2022	60°11'03.0"N 24°50'16.9"E	GG115	445	4689	0,094903	5,1	cf *Dolichospermum*, cf *Nodularia*
51	Otaniemi	24.8.2022	60°11'10.7"N 24°50'17.2"E	GG125	307	5318	0,0577285	4,9	Cells broken, fragments hard to identify
52	Siltatie,Kemiö	25.8.2022	60°09'13.4"N 22°36'50.5"E	GG127	6	123	0,0487805	4,9	Cells broken, fragments hard to identify
53	Dragsfjärd	25.8.2022	60°04'24.3"N 22°28'02.3"E	GG143	1317	2555	0,5154599	5,1	cf *Dolichospermum*, cf *Aphanizomenon*
54	Dalsbruk	25.8.2022	60°01'10.9"N 22°30'13.0"E	GG136	19	1554	0,0122265	4,9	cf *Nodularia*, other broken cell fragments
55	Sundvik	25.8.2022	60°03'48.2"N 22°46'02.5"E	GG138	623	3560	0,175	5	cf *Aphanizomenon*, cf *Nodularia,* cf *Dolichospermum*
56	Finby	25.8.2022	60°06'48.8"N 22°57'24.9"E	GG140	1660	6002	0,2765745	4,9	cf *Nodularia*, other broken cell fragments: cf *Aphanizomenon*
57	Laajalahti	24.8.2022	60°12'11.0"N 24°49'45.2"E	GG144	119	1409	0,0844571	5,6	cf *Aphanizomenon*, cf *Nodularia*
58	Merimasku	30.8.2022	60°28'58.2"N 21°52'15.5"E	GG147	62	1241	0,0499597	5,4	cf *Aphanizomenon,* cf *Dolichospermum,* cf *Nodularia*
59	Naantali	30.8.2022	60°28'11.3"N 22°00'56.3"E	GG151	10	233	0,0429185	5,2	cf *Aphanizomenon,* cf *Dolichospermum*

Table S2. Strain number, origin, and assembly and descriptive statistics for the draft and complete genomes of *Anabaena*, *Dolichospermum* and *Aphanizomenon* used in the phylogenomic analysis.

Strain	**Location**	**Assembly**	**Completeness (%)**	**Contamination (%)**	**Contigs**	**Largest contig**	**Total length**	**GC (%)**	**N50**	**N75**	**L50**	**L75**	**# N's per 100 kbp**
Anabaena aphanizomenioides LEGE 00250	Europe	GCA_015207835_1	98.78	0.22	399	112128	5585676	37.59	23683	13073	67	147	0
Anabaena azotica FACHB119	Asia	GCA_014697625_1	99.56	0.89	122	311233	8048170	41.34	127363	79827	21	42	0
Anabaena catenula FACHB362	Asia	GCA_014698735_1	99.89	0.00	109	435731	6234385	39.37	120473	57818	18	36	0
Anabaena cylindrica FACHB170	Asia	GCA_014696775_1	99.19	0.00	71	723181	7003498	41.26	208506	96332	10	21	0
Anabaena cylindrica FACHB243	Asia	GCA_014697335_1	99.44	0.00	96	418195	6890297	38.77	143668	78453	16	31	0
Anabaena cylindrica FACHB318	Asia	GCA_014696465_1	99.07	0.00	73	726430	6957411	41.27	208452	90174	11	23	0
Anabaena cylindrica PCC7122	Europe	GCA_000317695_1	99.44	0.00	7	6395836	7063285	38.79	6395836	6395836	1	1	0
Anabaena cylindrica UHCC 0172	Europe	GCA_034931625.1	99.15	0.00	154	195863	6371687	38.66	66312	39261	33	63	28.25
Anabaena lutea FACHB196	Asia	GCA_014698305_1	99.67	0.00	63	529190	6059416	39.25	192513	109373	10	21	0
Anabaena minutissima FACHB250	Asia	GCA_014697125_1	99.33	0.00	119	597336	7065205	41.08	161999	74121	14	31	0
Anabaena sp. 4–3	North America	GCA_001597745_1	98.89	0.22	62	496013	5598787	41.4	206377	129956	10	19	105.4
Anabaena sp. 49628 E55	North America	GCA_021300315_1	99.22	0.00	112	215540	4873444	37.29	79416	45159	21	40	6.5
Anabaena sp. 49633 E8	North America	GCA_021300225_1	99.56	0.22	318	223937	8566965	38.07	38235	21334	69	144	4.13
Anabaena sp. 54	Europe	GCA_017355725_1	99.11	0.45	69	482238	5567125	38.33	122796	73166	12	26	0
Anabaena sp. 90	Europe	GCA_000312705_1	91.38	77.41	5	4329264	5305675	38.1	4329264	4329264	1	1	0
Anabaena sp. AL09	North America	GCA_001672255_1	98.33	0.19	109	261741	4657189	38.14	64976	38711	23	46	94.2
Anabaena sp. AL93	North America	GCA_001672085_1	99.67	0.00	217	133848	5662506	38.36	46343	28501	44	83	0
Anabaena sp. CA ATCC 33047	North America	GCA_001597855_1	98.11	0.00	67	578591	5549443	41.39	201628	114799	9	18	230.04
Anabaena sp. CoA2 C59	North America	GCA_021297915_1	99.67	0.52	162	111287	4225696	37.03	41432	22791	35	69	0
Anabaena sp. CRKS33	North America	GCA_001672075_1	98.56	0.22	865	55883	4860204	37.52	13640	6103	105	238	0
Anabaena sp. FACHB1237	Asia	GCA_014696225_1	99.44	0.22	95	224844	4647682	34.96	97967	43396	15	32	0
Anabaena sp. FACHB1250	Asia	GCA_014696335_1	99.67	1.78	107	170919	4436573	37.64	72652	43942	23	42	0
Anabaena sp. FACHB1391	Asia	GCA_014696755_1	98.67	0.00	90	208994	4497192	37.64	102015	57036	17	32	0

Anabaena sp. FACHB709	Asia	GCA_014696765_1	99.00	0.33	80	657653	7102143	41.26	205481	89988	12	24	0
Anabaena sp. FACHB83	Asia	GCA_014697485_1	99.22	0.33	116	516073	8511586	40.64	190745	73849	14	32	0
Anabaena sphaerica FACHB251	Asia	GCA_014696825_1	99.19	0.00	46	846047	6198606	38.79	296026	132715	6	15	0
Anabaena sp. LE011 02	North America	GCA_001672225_1	99.48	0.11	122	215935	4740750	38.08	64747	33199	23	49	45.06
Anabaena sp. MDT14b	North America	GCA_001672195_1	99.22	0.11	1059	34317	4890627	38.74	8249	4605	176	376	0
Anabaena sp. PCC7108	Europe	GCA_000332135_1	97.17	4.22	3	5714552	5886741	38.78	5714552	5714552	1	1	16.99
Anabaena sp. SpSt 373	North America	GCA_011332035_1	99.63	0.07	153	244587	7193660	40.87	83043	49100	29	57	0
Anabaena sp. UBA12330	North America	GCA_003525565_1	99.56	0.44	81	204597	4285598	36.99	90280	54352	18	34	357.8
Anabaena sp. UHCC 0204	Europe	GCA_009711975_1	98.56	0.22	83	344565	5744555	37.57	131886	78143	13	26	161.04
Anabaena sp. UHCC 0253	Europe	GCA_009712085_1	99.22	0.22	28	945358	6116306	37.55	553243	326530	4	8	1475.11
Anabaena sp. UHCC 0399	Europe	GCA_034931465.1	97.67	0.44	118	229763	6570125	41.04	90169	60776	24	46	117.32
Anabaena sp. UHCC 0451	Europe	GCA_034931405.1	99.78	0.22	74	348687	5621773	37.86	140240	80181	15	27	306.61
Anabaena sp. WA102	North America	GCA_001277295_1	99.89	0.44	2	5705437	5782034	38.38	5705437	5705437	1	1	0
Anabaena sp. WA113	North America	GCA_001672155_1	99.33	0.00	261	172747	4687987	37.23	71945	35358	22	44	0
Anabaena sp. YBS01	Asia	GCA_009498015_1	99.22	0.00	1	7013200	7013200	41.43	7013200	7013200	1	1	0
Anabaena subtropica FACHB260	Asia	GCA_014697105_1	99.33	0.22	105	342035	5809825	40.83	108475	56412	17	36	0
Anabaena variabilis FACHB164	Asia	GCA_014698355_1	99.67	0.00	33	648438	5986679	39.32	394797	243352	6	11	0
Anabaena variabilis FACHB171	Asia	GCA_014697155_1	99.33	0.22	87	478819	7067220	41.27	185514	89988	13	26	0
Aphanizomenon flos aquae 2012KM1D3	Europe	GCA_000789435_1	87.52	7.22	325	120451	5741771	38.22	25535	14018	73	148	0.47
Aphanizomenon flos aquae CCAP 14461C	North America	GCA_019800585_1	98.11	0.00	286	181707	6756227	38.75	37602	20260	53	114	17.11
Aphanizomenon flos aquae CCAP 14461C	North America	GCA_019800605_1	98.33	0.00	254	177696	6761576	38.75	43156	24189	49	100	24.57
Aphanizomenon flos aquae Clear A1	North America	GCA_014654815_1	99.89	0.22	60	381826	4442226	37.14	120880	84307	11	23	56.28
Aphanizomenon flos aquae CP01	North America	GCA_017355665_1	99.22	0.89	152	295745	4356503	36.95	54035	33983	22	46	0
Aphanizomenon flos aquae DEX188	North America	GCA_017346855_1	99.89	0.00	1	4538078	4538078	37.06	4538078	4538078	1	1	0
Aphanizomenon flos aquae	Asia	GCA_014696815_1	99.22	0.11	155	163255	5786747	38.4	58342	37171	31	62	0

FACHB1040													
Aphanizomenon flos aquae FACHB1171	Asia	GCA_014697315_1	99.44	0.00	91	214077	4343146	37.04	78897	47174	19	36	0
Aphanizomenon flos aquae FACHB1249	Asia	GCA_014698725_1	99.89	0.00	97	321580	4350034	37.01	83039	44916	18	35	0
Aphanizomenon flos aquae FACHB1265	Asia	GCA_014698705_1	99.89	0.00	88	223715	4343226	37.02	85704	41837	16	34	0
Aphanizomenon flos aquae FACHB1287	Asia	GCA_014698755_1	99.89	0.00	85	223784	4347270	37.03	85728	49088	16	33	0
Aphanizomenon flos aquae FACHB1290	Asia	GCA_014698295_1	99.67	0.22	136	156477	4314391	37.01	55341	28108	26	55	0
Aphanizomenon flos aquae FACHB1416	Asia	GCA_014698695_1	99.89	0.22	162	137305	4287223	37.13	43168	25223	32	62	0
Aphanizomenon flos aquae KM1D3 PB	Europe	GCA_017346875_1	99.44	0.22	1	6071322	6071322	38.39	6071322	6071322	1	1	0
Aphanizomenon flos aquae LD13	North America	GCA_001672165_1	99.67	0.37	199	160417	4401776	37.03	55961	29540	27	53	0
Aphanizomenon flos aquae SRR3727505	Europe	Unpublished data	99.00	1.00	399	50419	4132940	37.09	14233	8522	93	186	102.83
Aphanizomenon flos aquae MDT14a	North America	GCA_001672095_1	99.44	0.56	171	242078	4623430	37.09	60437	33975	27	52	0
Aphanizomenon flos aquae NIES-81	Asia	GCA_000521175_1	99.67	0.22	38	2605121	5831251	38.47	1857322	1857322	2	2	2894.34
Aphanizomenon flos aquae UKL13 PB	North America	GCA_001593825_2	99.89	3.60	15	958282	4484012	37.04	767568	398059	3	5	0
Aphanizomenon flos aquae WA102	North America	GCA_001672105_1	98.48	11.36	1106	119846	5923586	39.11	24228	11348	68	154	0
Aphanizomenon sp. FACHB1399	Asia	GCA_014698245_1	99.67	0.00	100	331699	4348078	37.01	76250	42563	19	37	0
Aphanizomenon sp. FACHB1401	Asia	GCA_014698265_1	99.67	0.00	104	153821	4349063	37.05	75924	41787	21	40	0
Aphanizomenon sp. UHCC 0183	Europe	GCA_009712065_1	99.22	0.22	182	177534	5579684	38.36	56985	26535	31	69	593.74
Chroococcidiopsis thermalis PCC 7203	Europe	GCA_000317125_1	99.63	1.44	3	6315792	6689401	44.47	6315792	6315792	1	1	0
Cuspidothrix issatschenkoi CHARLIE1	Europe	GCA_002934005_1	97.78	0.00	243	160023	4547138	37.67	35946	19639	42	87	93.25
Cuspidothrix issatschenkoi LEGE	Europe	GCA_015207795_1	100.00	0.11	333	97409	5113591	37.75	29781	15454	55	111	0.06

03284													
Dolichospermum circinale AWQC131C	Oceania	GCA_000426905_1	99.78	0.00	121	254267	4445648	37.33	80443	52884	17	34	867.21
Dolichospermum circinale AWQC310F	Oceania	GCA_000426925_1	99.78	0.00	82	360115	4405846	37.49	119350	65648	13	25	425.25
Dolichospermum circinale Clear D4	North America	GCA_014654715_1	92.05	6.49	560	117237	5029933	36.96	18484	6545	75	192	224.66
Dolichospermum compactum NIES806	Asia	GCA_002368115_1	99.33	0.22	1	5165988	5165988	38.06	5165988	5165988	1	1	1587.63
Dolichospermum flos aquae CCAP 140313F	North America	GCA_012516395_1	99.67	0.44	2	5151752	5220684	38.15	5151752	5151752	1	1	0
Dolichospermum flos aquae LEGE 04289	Europe	GCA_015207785_1	99.00	0.41	135	295537	4435098	37.65	54871	32276	27	54	0
Dolichospermum flos aquae UHCC 0037	Europe	GCA_009712125_1	99.00	0.63	9	1818114	5563105	38.33	1095036	613144	2	4	136.74
Dolichospermum planctonicum NIES-80	Asia	GCA_005402965_1	99.22	0.33	152	221952	4556907	37.7	75157	45488	20	39	0
Dolichospermum planctonicum UHCC 0167	Europe	GCA_009712075_1	97.55	0.19	33	564450	4900328	37.47	253071	206132	7	12	3391.04
Dolichospermum sp. BR01	North America	GCA_018447705_1	99.89	4.11	3	5439257	5575959	38.19	5439257	5439257	1	1	0
Dolichospermum sp. DET50	North America	GCA_018295975_1	99.67	0.22	3	5825454	6065531	37.8	5825454	5825454	1	1	0
Dolichospermum sp. DET66	North America	GCA_018295985_1	99.67	0.00	3	5835936	6113795	37.79	5835936	5835936	1	1	0
Dolichospermum sp. DET67	North America	GCA_018295965_1	99.67	0.00	5	5835936	6126501	37.79	5835936	5835936	1	1	0
Dolichospermum sp. DET69	North America	GCA_017355425_1	99.67	0.00	2	5839262	6040057	37.8	5839262	5839262	1	1	0
Dolichospermum sp. DET73	North America	GCA_017355625_1	99.67	0.00	286	120710	5233469	38.06	31278	16316	56	113	0
Dolichospermum sp. DEX182a	North America	GCA_017355645_1	99.67	1.11	4	3410753	5014232	38.29	3410753	1583037	1	2	0
Dolichospermum sp. DEX189	North America	GCA_017355655_1	98.22	1.11	8	3703826	6054507	38.39	3703826	895365	1	2	0
Dolichospermum sp. DL01	North America	GCA_017346835_1	97.67	0.11	1	4827232	4827232	38.19	4827232	4827232	1	1	0
Dolichospermum sp. FACHB1091	Asia	GCA_014697585_1	99.67	0.00	59	252710	4516276	37.5	124205	71273	13	25	0
Dolichospermum sp. JUN01	North America	GCA_017355635_1	99.55	0.45	253	234876	5023336	38.33	75344	31935	24	50	0
Dolichospermum sp. LBC05a	North America	GCA_017346815_1	94.19	3.74	1	5178591	5178591	38.21	5178591	5178591	1	1	0

Dolichospermum sp. LEGE 00240	Europe	GCA_015207875_1	99.67	0.11	172	201700	5192325	38.42	55172	30510	29	60	0
Dolichospermum sp. LEGE 00246	Europe	GCA_015207865_1	100.00	0.96	779	48182	5708300	38.77	10796	5958	162	340	0
Dolichospermum sp. OL01	North America	GCA_018447755_1	98.33	0.44	2	5178840	5274066	38.19	5178840	5178840	1	1	0.02
Dolichospermum sp. SB001	North America	GCA_016462165_1	99.67	0.11	965	24387	4311702	38.35	5456	3411	254	505	184.85
Dolichospermum sp. UHCC 0187	Europe	GCA_009712035.1	90.96	0.56	76	759950	5308777	38.2	161299	84937	8	19	88.34
Dolichospermum sp. UHCC 0259	Europe	GCA_009711935_1	99.89	0.67	316	181958	5630158	38	34021	18346	50	108	30.34
Dolichospermum sp. UHCC 0260	Europe	GCA_009711985_1	99.22	0.00	113	184152	4921436	38.09	74043	38012	23	45	603.67
Dolichospermum sp. UHCC 0299	Europe	GCA_009711965_1	99.67	0.22	288	160154	5118028	38.05	31633	17653	52	106	8.11
Dolichospermum sp. UHCC 0315A	Europe	GCA_008121535_1	99.67	0.22	4	5407479	5642022	38.16	5407479	5407479	1	1	0
Dolichospermum sp. UHCC 0352	Europe	GCA_009711925_1	99.44	0.00	314	88234	5122584	38.01	31029	16542	54	110	32.89
Dolichospermum sp. UHCC 0406	Europe	GCA_009712025_1	99.22	0.22	42	515837	5178080	38.04	354243	181847	6	12	109.21
Dolichospermum sp. UHCC 0684	Europe	GCA_034931665.1	96.59	0.00	274	83516	4841461	38.05	27888	14602	57	116	121.86
Dolichospermum sp. UKL201	North America	GCA_017346795_1	99.67	0.00	1	5294355	5294355	38.2	5294355	5294355	1	1	0
Dolichospermum sp. WA123	North America	GCA_018447775_1	99.67	0.00	3	4881389	4984963	38.28	4881389	4881389	1	1	0

Table S3. Number of strains, MAA biosynthetic gene clusters and the relative abundance of MAA biosynthetic gene clusters in the genomes of 101 analyzed *Anabaena*, *Dolichospermum*, *Aphanizomenon* (ADA) strains.

**Species/group**	**Number of strains**	**Number of MAA biosynthetic gene clusters**	**Relative abundance (%)**
ADA-1	10	0	0.00
ADA-2	22	13	59.09
ADA-3	11	3	27.27
ADA-4	19	19	100.00
ADA-5	1	0	0.00
ADA-6	4	0	0.00
ADA-7	2	1	50.00
ADA-8	2	0	0.00
ADA-9	1	0	0.00
ADA-10	1	0	0.00
ADA-11	1	0	0.00
ADA-12	1	0	0.00
All ADA	75	36	48.00
Others	26	11	42.30
All	101	47	46.53

Table S4. The strain number, year of isolation, growth medium, and sampling location for the *Dolichospermum* strains (Halinen *et al*., 2007 and Halinen *et al*., 2008). Z8 growth medium (Kotai, 1972) *supplemented with NaCl and $MgSO_4$. $^{+}$Approximated coordinates. N/A indicates coordinates not available.

**UHCC code**	**Strain**	**Year**	**Suppl***	**Location**	**Coordinates**	**MAA**
UHCC 0652	BIR3	2003	+	The Gulf of Finland	60.10, 26.15^{+}	-
UHCC 0762	BIR4	2003	+	The Gulf of Finland	60.10, 26.15^{+}	-
UHCC 0680	BIR25	2004	-	The Gulf of Finland	59.2901, 23.5702	-
UHCC 0631	BIR28	2004	-	The Gulf of Finland	59.2901, 23.5702	-
UHCC 0626	BIR30	2004	+	The Gulf of Finland	59.2901, 23.5702	-
UHCC 0310	BIR49	2004	+	The Gulf of Finland	59.2901, 23.5702	-
UHCC 0650	BIR50	2004	-	The Gulf of Finland	59.5701, 24.4500	+
UHCC 0627	BIR50	2004	+	The Gulf of Finland	59.3515, 24.0700	-
UHCC 0640	BIR52	2004	-	The Gulf of Finland	59.3515, 24.0700	+
UHCC 0639	BIR53	2004	-	The Gulf of Finland	59.3515, 24.0700	-
UHCC 0641	BIR56	2004	-	The Gulf of Finland	59.3515, 24.0700	-
UHCC 0656	BIR66	2004	-	The Gulf of Finland	59.2551, 22.5206	-
UHCC 0643	BIR73	2004	-	The Gulf of Finland	59.5701, 24.4500	-
UHCC 0638	BIR78	2004	-	The Gulf of Finland	59.5701, 24.4500	-
UHCC 0658	BIR84	2004	-	The Gulf of Finland	59.2551, 22.5206	-
UHCC 0671	BIR94	2004	-	The Gulf of Finland	59.3882, 23.3903	-
UHCC 0661	BIR130	2004	-	The Gulf of Finland	59.3020, 22.4031	-
UHCC 0663	BIR162	2004	-	The Gulf of Finland	59.1259, 22.1894	-
UHCC 0753	BIR169	2004	-	The Gulf of Finland	59.1259, 22.1894	-
UHCC 0291	BIR202	2004	+	The Gulf of Finland	59.3250, 23.1000	-

UHCC 0676	BIR208	2004	-	The Gulf of Finland	59.3250, 23.1000	-
UHCC 0620	BIR232	2004	+	The Gulf of Finland	59.2802, 22.3858	-
UHCC 0677	BIR241	2004	-	The Gulf of Finland	N/A	-
UHCC 0767	BIR246	2004	-	The Gulf of Finland	60.0400, 26.2087	+
UHCC 0684	BIR258	2004	-	The Gulf of Finland	60.0400, 26.2087	+
UHCC 0259	BIR259	2004	-	The Gulf of Finland	60.0400, 26.2087	-
UHCC 0260	BIR260	2004	-	The Gulf of Finland	60.0400, 26.2087	+
UHCC 0629	BIR272	2004	-	The Gulf of Finland	59.3218, 22.5014	-
UHCC 0624	BIR348	2004	+	The Gulf of Finland	59.2400, 22.2551	-
UHCC 0623	BIR358	2004	+	The Gulf of Finland	59.2400, 22.2551	-
UHCC 0673	BIR370A	2004	-	The Gulf of Finland	59.1330, 22.2851	-
UHCC 0633	BIR370B	2004	-	The Gulf of Finland	59.1330, 22.2851	-
UHCC 0634	BIR374	2004	-	The Gulf of Finland	59.1701, 22.4700	-
UHCC 0625	BIR406	2004	+	The Gulf of Finland	59.4181, 23.5798	-
UHCC 0635	BIR441	2004	-	The Gulf of Finland	59.3515, 24.0700	-
UHCC 0315	315	1997	-	Seurasaari, Finland	N/A	

Table S5. The relative abundances (%) of porphyra-334 and shinorine detected at 334 nm in five strains of *Dolichospermum* normalized against *Dolichospermum* sp. UHCC 0684. The culture producing higher relative abundance of MAAs were used for LC/MS-QTOF analysis and are highlighted in green.

**MAA**	**UHCC 0650**	**UHCC 0640**	**UHCC 0767**	**UHCC 0684**	**UHCC 0260**
Porphyra-334	3.5	10	0.5	63.5	22.5
Shinorine	7	15.7	1.3	36.5	39.6

Table S6. The experienced mass-to-charge ratio (m/z), ion intensity, theoretical mass-to-charge ratio (*m/z*), mass error (ppm), molecular formular and putative fragmentations of porphyra-334 (*m/z* 347.1449) and shinorine.

	Porphyra-334, R = CH_3				Shinorine, R = H			
No	Molecular formula	Theoretical (m/z)	Δ (ppm)	I (x 1000)	Theoretical (m/z)	Δ (ppm)	I (x 100)	Neutral loss
1	$C_{14}H_{23}N_2O_8^{+}$	347.14489	-1.3	195	333.12924	-2.1	88	N/A
2	$C_{13}H_{20}N_2O_8^{\bullet+}$	332.12142	-1.7	3	318.10577	-11.7	2	d
3	$C_{13}H_{18}N_2O_7^{\bullet+}$	314.11085	-1.0	7	300.09520	4.2	9	d, H_2O
4	$C_{12}H_{19}N_2O_7^{+}$	303.11868	-0.8	147	303.11868	-0.4	6	k
5	$C_{12}H_{19}N_2O_6^{+}$	301.13941	0.1	9	287.12376	3.8	8	m
6	$C_{13}H_{16}N_2O_6^{\bullet+}$	296.10029	-1.8	7	282.08464	3.2	8	d, 2xH_2O
7	$C_{12}H_{20}N_2O_6^{\bullet+}$	288.13159	-1.5	11	274.11594	1.5	10	a/m, d
8	$C_{11}H_{15}N_2O_7^{+}$	287.08738	-0.8	19	287.08738	-2.9	6	d, k
9	$C_{13}H_{15}N_2O_5^{+}$	279.09755	-2.5	10	265.08190	-2.8	15	f, g, l
10	$C_{12}H_{18}N_2O_5^{\bullet+}$	270.12102	-1.4	10	256.10537	-9.4[a]	19	a, d, H_2O
11	$C_{12}H_{17}N_2O_5^{+}$	269.11320	-3.5	9	255.09755	0.4	50	e, f, g
12	$C_{12}H_{16}N_2O_5^{\bullet+}$	268.10537	-3.4	8	254.08972	1.7	8	e, f, g
13	$C_{11}H_{20}N_2O_4^{\bullet+}$	244.14176	-2.9	27	230.12611	-0.3	25	a, d, m
14	$C_{10}H_{16}N_2O_5^{\bullet+}$	244.10537	-4.6	20	244.10537	-5.8	4	a/m, d, k
15	$C_{10}H_{15}N_2O_5^{+}$	243.09755	-3.3	37	243.09755	-3.7	12	e, g, k
16	$C_{10}H_{13}N_2O_5^{+}$	241.08190	-1.5	25	241.08190	1.5	19	e, g, k
17	$C_{10}H_{15}N_2O_4^{+}$	227.10263	0.5	31	227.10263	1.4	6	(j, H_2O)/(b, k, H_2O)/(a/m, g, k)
18	$C_{10}H_{13}N_2O_3^{+}$	209.09207	1.8	29	209.09207	2.8	12	a/m, f, g, k
19	$C_9H_{16}N_2O_3^{\bullet+}$	200.11554	-0.4	116	200.11554	4.5	16	a, d, k, m
20	$C_9H_{15}N_2O_3^{+}$	199.10772	-0.9	49	199.10772	5.2	24	a, d, k, m
21	$C_9H_{13}N_2O_3^{+}$	197.09207	-0.6	71	197.09207	-2.6	56	e, f, j
22	$C_9H_{11}N_2O_3^{+}$	195.07642	2.7	29	195.07642	5.8	12	f, g, j
23	$C_8H_{14}N_2O_3^{\bullet+}$	186.09989	1.9	95	186.09989	0.9	64	a, d, j
24	$C_8H_{13}N_2O_3^{+}$	185.09207	1.5	40	185.09207	-1.7	9	e, g, j
25	$C_9H_{11}N_2O_2^{+}$	179.08150	0.8	23	179.08150	0.8	12	(a/m, e, f, g, k)/(b, e, f, h, k)/(e, f, h, j)
26	$C_8H_{10}NO_3^{+}$	168.06552	2.0	28	168.06552	-1.0	11	e, g, i
27	$C_9H_{11}N_2O^{+}$	163.08659	2.2	23	163.08659	-3.9	16	a/m, f, g, k
28	$C_8H_{11}N_2O^{+}$	151.08659	2.4	34	151.08659	-3.6	15	(a, j)/(b, k, m), f, g
29	$C_8H_9N_2O^{+}$	149.07094	5.4	61	149.07094	5.4	21	(b, f, g, k, m)/(a, f, g, j)/(b, f, h, j)
30	$C_7H_9N_2O^{+}$	137.07094	4.5	52	137.07094	3.7	23	b, f, g, j
31	$C_7H_8NO^{+}$	122.06004	5.0	20	122.06004	5.8	8	(b, i)/(c, j), f, g
32	$C_6H_8NO^{+}$	110.06004	4.6	13	110.06004	4.6	5	(b, i)/(c, j), e, g
33	$C_7H_8N^{+}$	106.06512	2.2	13	106.06512	6.9	4	a/(k,m), e, f, g, i

Table S7. The observed retention times and peak areas of compounds with absorption maxima at 334 nm detected from the 36 analyzed *Dolichospermum* strains in the UPLC analysis. **A**. Compound assigned tentatively as porphyra-334. **B**. Compound assigned tentatively as shinorine.

	A. Porphyra-334		B. Shinorine	
**Strain**	**Retention time (min)**	**Peak area**	**Retention time (min)**	**Peak area**
**UHCC 0684**	6.29	14470.504	6.71	400.688
**UHCC 0260**	6.29	5127.837	6.71	436.244

Table S8. The maximum absorption, molecular weights, mass of protonated molecules, molar extinction coefficients and molecular formulas of porphyra-334 and shinorine (as reported in La Barre *et al*., 2014).

MAA	Absorbance λmax (nm)	Molecular weight (g/mol)	$[M + H]^+$ (*m/z*)	Extinction coefficient ε (L/mol/cm)	Molecular formula
**Porphyra-334**	334	346.336	347.1	42300	$C_{14}H_{22}N_2O_8$
**Shinorine**	334	332.31	333.1	44668	$C_{13}H_{20}N_2O_8$

Table S9. Obtained adjusted p values from the Kruskal–Wallis tests with post hoc Dunn's test.

Comparison	Adjusted *p*
2021 Helsinki vs. 2021 Turku	2.734166e-01
2021 Helsinki vs. 2021 Helsinki	1.737499e-04
2021 Turku vs. 2022 Helsinki	2.659849e-05
2021 Helsinki vs. 2022 Turku	2.827127e-03
2021 Turku vs. 2022 Turku	3.263397e-04
2022 Helsinki vs. 2022 Turku	7.591861e-01

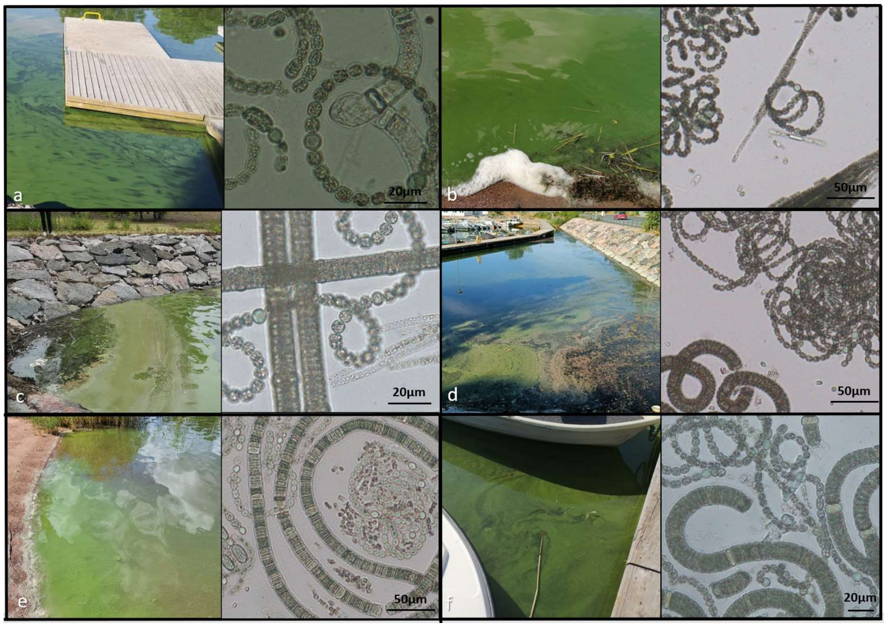

Figure S1. Blooms samples and microscopy images. (**a**) Rajasaari, Helsinki, 08.07.2022 (**b**) Munkkiniemi, Helsinki 19.07.2022 (**c**) Rajasaari, Helsinki, 05.08.2022 (**d**) Rajasaari, Helsinki, 09.08.2022 (**e**) Kultaranta, Turku (09.08.2022) (f) Kaarina, Turku, 16.08.2022.

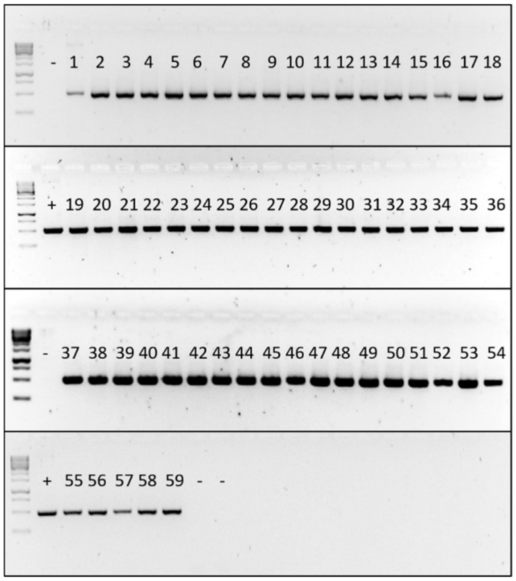

Figure S2. PCR amplification of *mysB* gene from 59 samples of surface blooms collected in 2021 and 2022. – indicates the negative control (DNAse/RNAse free water), + indicates the positive control (UHCC 0260).

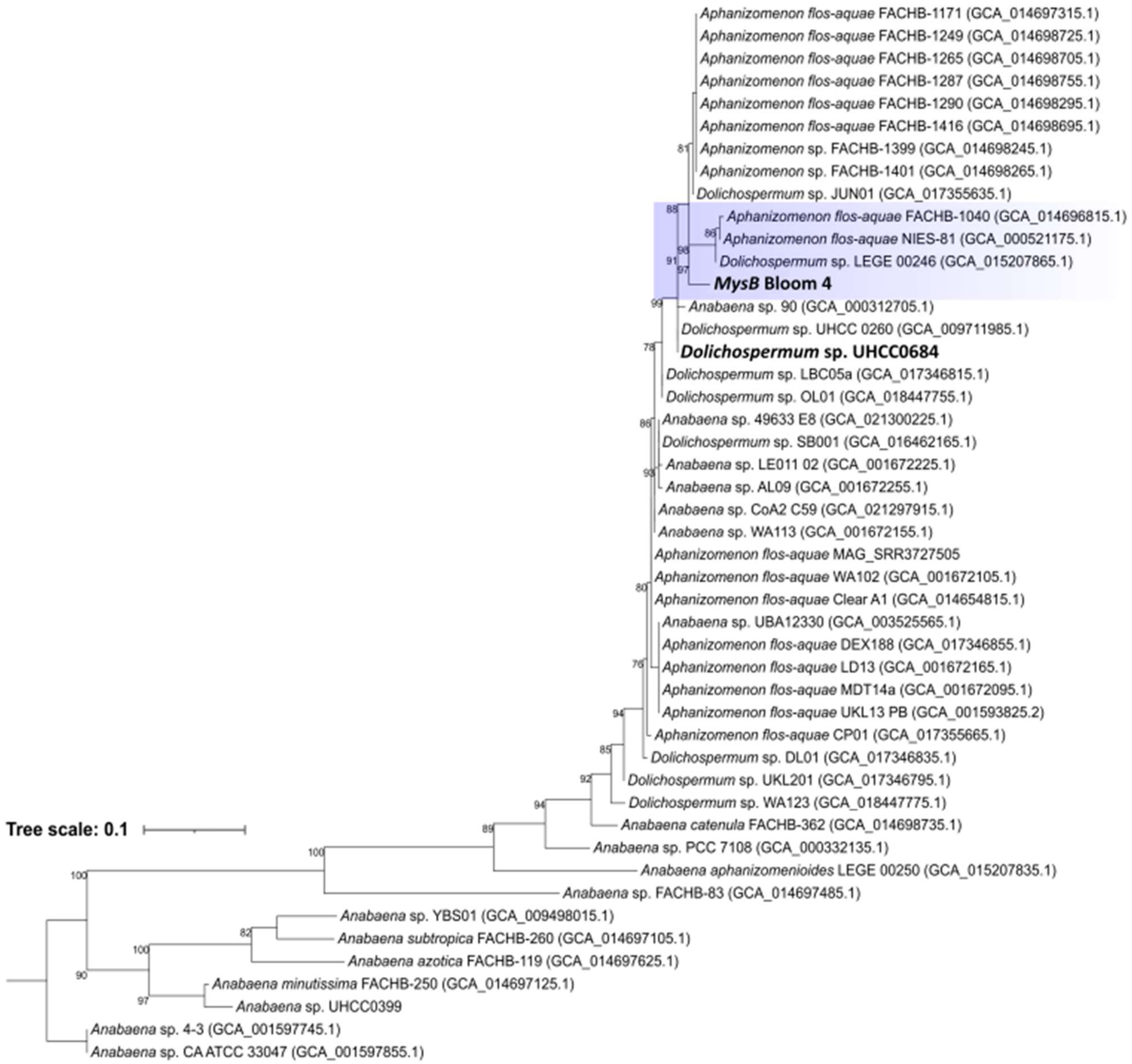

Figure S3. The phylogenetic tree of ADA clade showing position of the *mysB* gene amplified and sequenced from bloom sample no. 4.

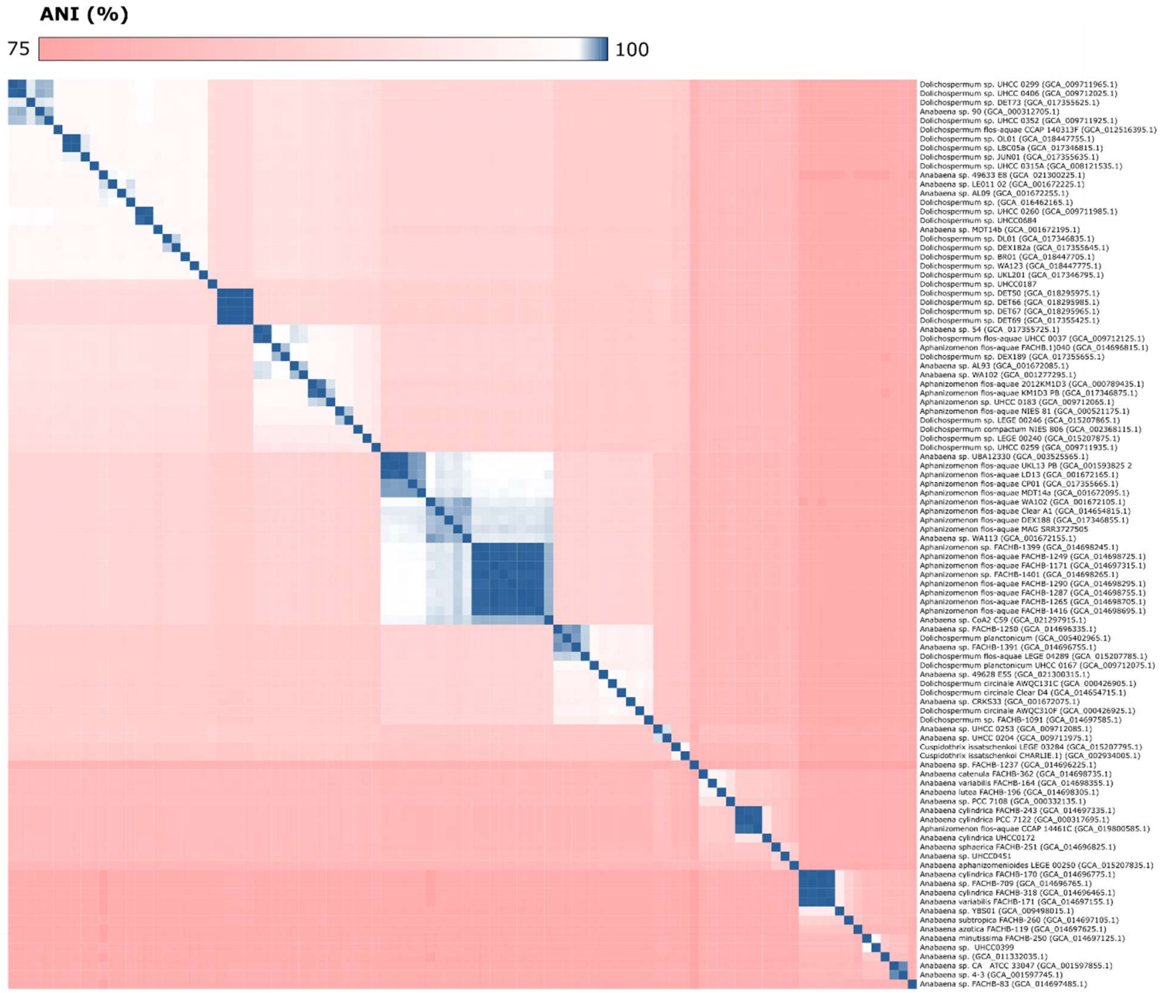

Figure S4. The Average Nucleotide Identities (ANI) of 101 *Anabaena*, *Dolichospermum* and *Aphanizomeon* complete of draft genomes analyzed (Table S4).

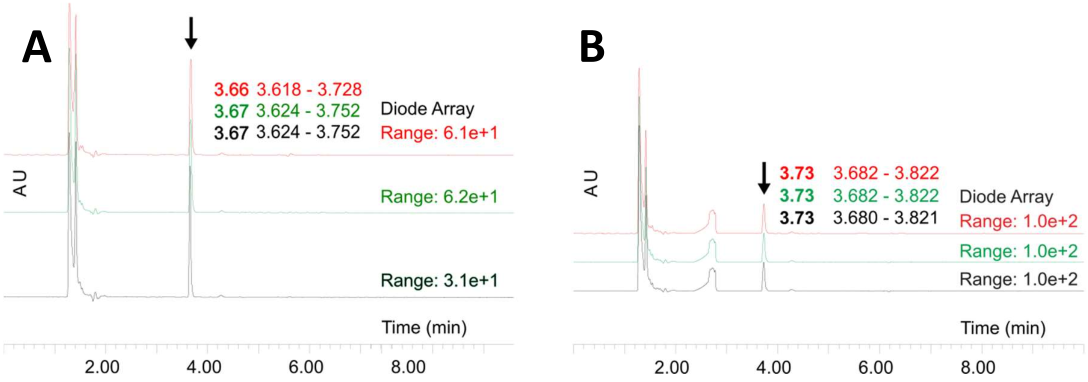

Figure S5. Collected porphyra-334 fractions from three injections of MAA methanol extracts from analyzed *Dolichospermum* samples. Collected peaks indicated by arrow, retention time (min) in bold and collected fraction (min) reported next to the peak. **A**. *Dolichospermum* sp. UHCC 0684, 1 µl injection in black, 2 µl injections in green and red. **B**. *Dolichospermum* sp. UHCC 0260, 1µl injection in black, 2 µl injections in green and red.

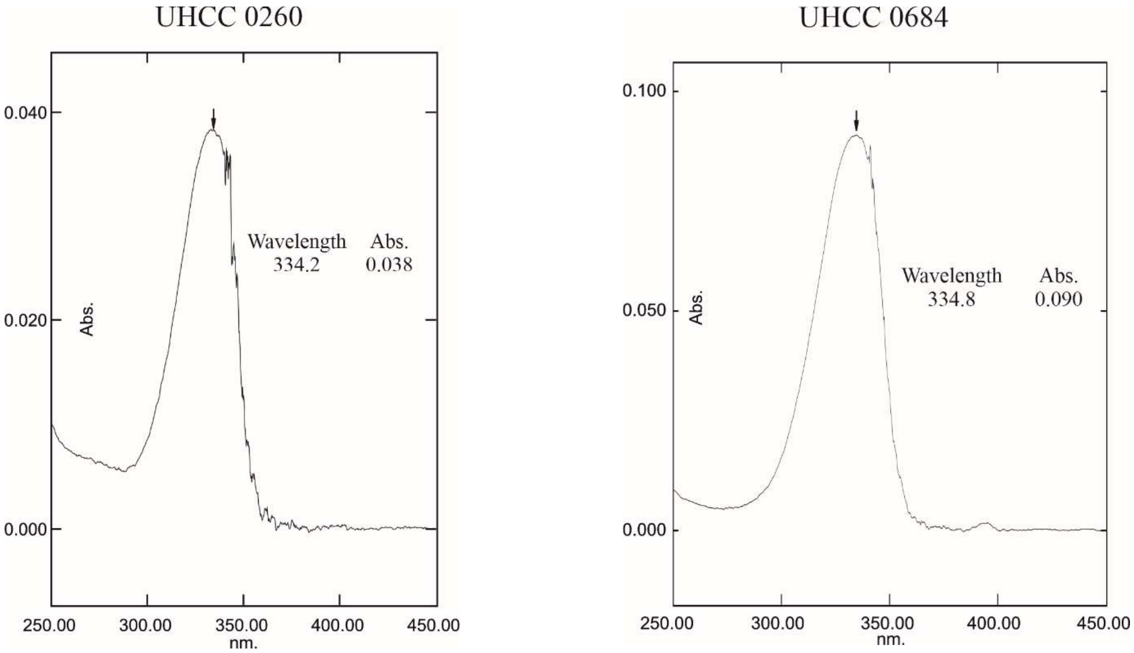

Figure S6. Purification of shinorine and porphyra-334 from *Dolichospermum* spp. UHCC 0260 and UHCC 0684.

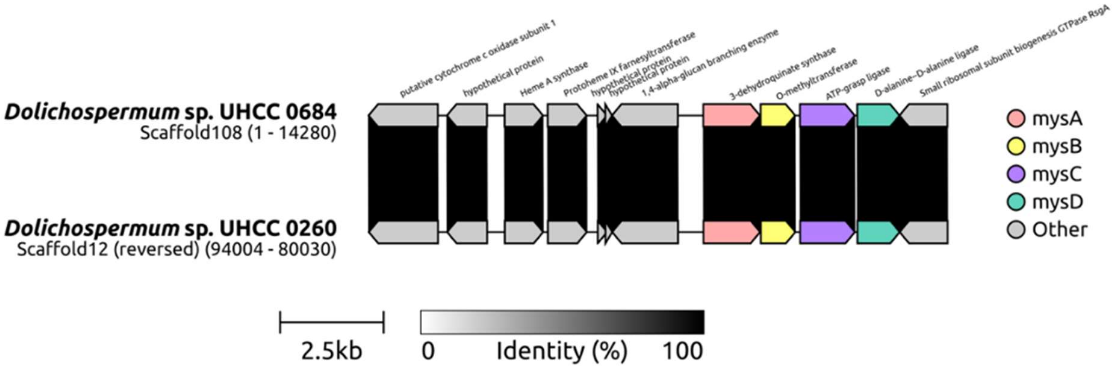

Figure S7. The comparison of MAA biosynthetic gene clusters from *Dolichospermum* spp. UHCC 0684 and UHCC 0260. The *mysA*-*mysB* genes and promoter region from *Dolichospermum* sp. UHCC 0684 and *Dolichospermum* sp. UHCC 0260 are 100 % identical at the nucleotide level.

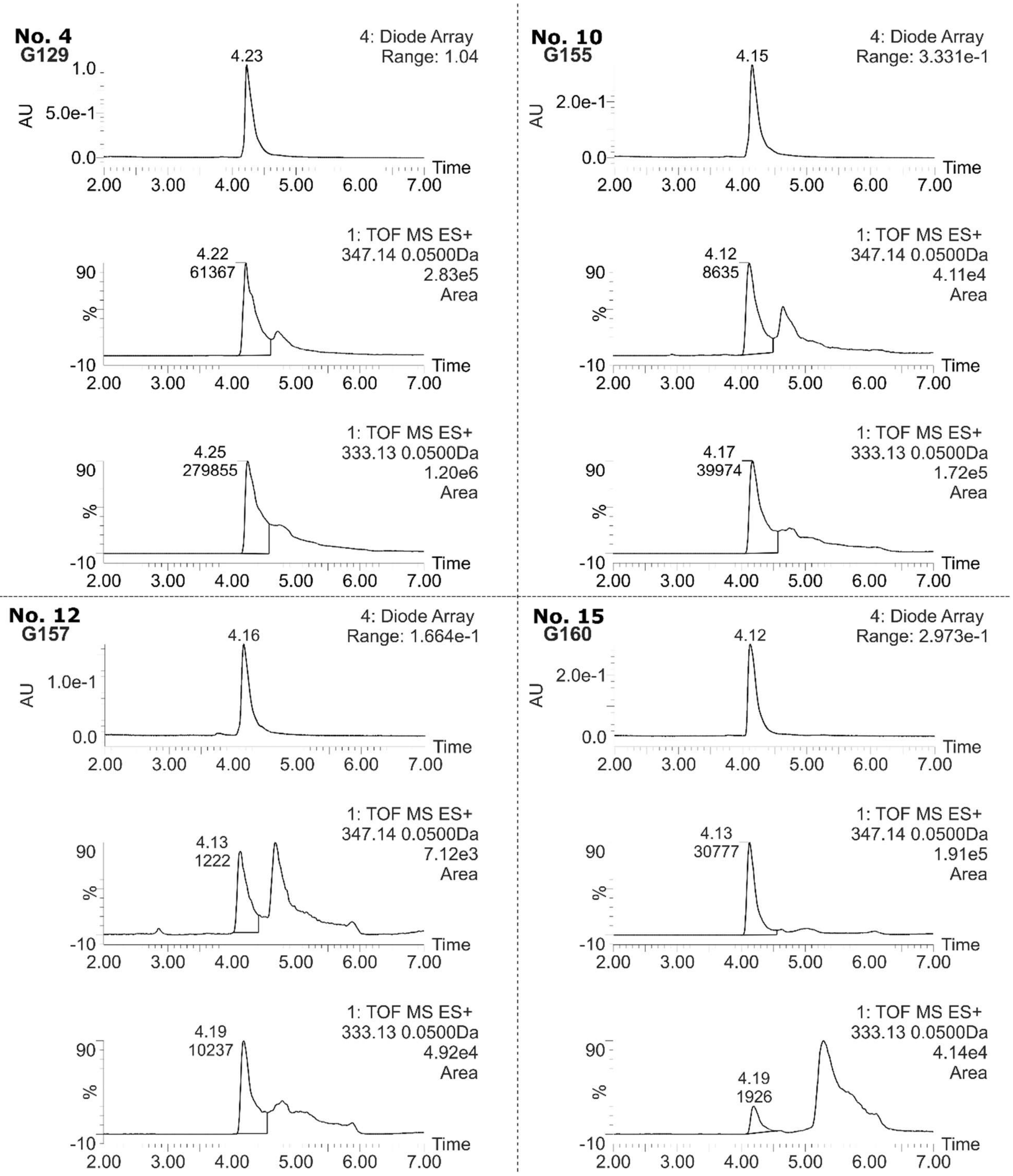

Figure S8. UV and MS ES+ chromatograms of the high MAA producing bloom samples no. 4, 10, 12 and 15 collected during the summer of 2021. (Porphyra-334: 347.14 Da, Shinorine: 333.13 Da)
